# Cytotoxic Activity of the Baltic Cyanobacterium *Pseudanabaena galeata* CCNP1313

**DOI:** 10.3390/toxins17120586

**Published:** 2025-12-06

**Authors:** Marta Cegłowska, Robert Konkel, Hanna Mazur-Marzec

**Affiliations:** 1 Marine Biochemistry Laboratory, Department of Marine Chemistry, Institute of Oceanology Polish Academy of Sciences, Powstańców Warszawy 55, PL-81712 Sopot, Poland; 2 Laboratory of Marine Biotechnology, Department of Marine Biology and Biotechnology, Faculty of Oceanography and Geography, University of Gdańsk, Marszałka Piłsudskiego 46, PL-81378 Gdynia, Poland; robert.konkel@ug.edu.pl

**Keywords:** Baltic *Pseudanabaena galeata*, cytotoxicity, bioactivity-based molecular networking

## Abstract

While tropical regions have traditionally been the focus of studies on natural bioactive products, works published within the last decade demonstrate that cyanobacteria from the Baltic Sea also possess significant biotechnological and pharmaceutical potential. The Baltic *Pseudanabaena galeata* CCNP1313 previously demonstrated activity against breast cancer cell lines (MCF7 and T47D) and several viruses. In the present study, the cytotoxicity of cellular extract and flash chromatography fractions from the strain were evaluated against a wider panel of cancer cells (A549, C-33A, CaSki, DoTC2, HeLa, PC3, SiHa, and T47D). To gain better insight into the compounds potentially responsible for the observed effects, high-resolution mass spectrometry was combined with bioactivity-based molecular networking. Both the extract and hydrophobic fractions showed strong cytotoxicity, particularly against breast cancer cells and selected cervical cancer cells. While HRMS analyses confirmed the production of previously characterised peptides by CCNP1313 (*Pseudanabaena galeata* peptides and galeapeptins), neither of them was found to be responsible for the activity. Instead, the molecular networking approach linked the cytotoxicity to specific lipid classes, including diacylglycerols (DAGs) and monogalactosyldiacylglycerols (MGDGs). This study highlights the necessity of integrating traditional methods with advanced bioinformatics for the successful discovery of bioactive natural products, especially when complex samples, such as extract or chromatographically separated fractions, are analysed.

## 1. Introduction

A comprehensive study by Popin et al. [1] investigated the presence and phylogenetic distribution of biosynthetic gene clusters (BGCs), which are associated with the biosynthesis of natural products, in 185 cyanobacterial strains. The authors found that individual cyanobacterial genomes contain between 1 and 42 BGCs. Their analyses also revealed that 95% of these clusters were located in chromosomes, while the remaining were plasmid-encoded. Furthermore, the number of BGCs was positively correlated with genome size. For example, cyanobacteria from the Synechococcales and Chroococcales orders were characterised by the smallest genomes (average 4.04 Mb) and the lowest number of BGCs [1]. The authors also investigated two *Pseudanabaena* genomes (5.28 ± 0.54 Mb), identifying three BGCs in the chromosome and one in the plasmid. In comparison, the Baltic *Pseudanabaena galeata* CCNP1313, which has a larger genome (5.84 Mb), possesses four BGCs located in the chromosome and one in the plasmid [2].

Most studies on *Pseudanabaena* have focused primarily on taxonomic and morphological characterisation, while information on metabolite production remains underexplored. Only a few studies have investigated the structure and bioactivity of metabolites encoded by *Pseudanabaena* BGCs. Among the identified compounds there were microcystin [3,4,5,6,7], odorous 2-methylisoborneol (2-MIB) [8,9,10], and various photosynthetic and accessory pigments [11,12,13,14,15]. The highest number of secondary metabolites (sixty-three) was reported from the Baltic *Pseudanabaena galeata* CCNP1313, among them ‘*Pseudanabaena galeata* peptides’ (PGs) and ‘galeatapeptins’ (GPs) were characterised [16]. In terms of bioactivity, the phycobiliprotein extract, from *P. tenuis*, reduced HgCl_2_-induced oxidative stress and cellular damage in renal cells [17]. Extracts from two *P. catenata* strains (MWQ8-11A, ATCC29207) were active against herpes simplex virus type 2 (HSV-2) [18]. Furthermore, Pseudanabaena strains from Portugal were cytotoxic against several cancer cell lines [19]. The cellular extract from the Baltic *P. galeata* CCNP1313 exhibited strong cytotoxic activity against breast cancer cells (MCF-7), and at the same time less significant effects were observed against cervical cancer cells (HeLa) [20]. In a subsequent study, the cytotoxicity and antiviral activity of cellular extracts, chromatographic fractions, and isolated peptides from CCNP1313 against one cancer cell line (breast cancer cells T47D), human coronavirus OC43 (HCoV-OC43), West Nile Virus (WNV), and severe acute respiratory syndrome coronavirus 2 (SARS-CoV-2) were explored [16]. The samples exhibited strong cytotoxicity against T47D cells and significantly inhibited viral replication (LRV −1.7 for WNV, −2.7 for HCoV-OC43, and from −0.3 to −1.8 for SARS-CoV-2) [16].

The World Health Organization (WHO) identifies cancer as a leading cause of mortality worldwide, with lung, breast, colorectal, and prostate cancers among the most frequently diagnosed. Among women, breast and cervical cancers are particularly prevalent and constitute major contributors to cancer-related deaths. Breast cancer alone accounts for nearly one in four cancer diagnoses and one in six cancer deaths among women globally. Cervical cancer also remains a substantial global health burden, with an estimated 660,000 new cases and 350,000 deaths reported in 2022 [21]. On the other hand, among men, lung and prostate cancer are most commonly diagnosed and are leading causes of cancer-related deaths. Prostate cancer, in particular, accounted for approximately 1,466,680 new cases and 396,792 deaths worldwide in 2022 [21]. Most current treatments are associated with adverse effects; therefore, safer and more effective therapies are needed. These considerations, together with the previously reported activity of *Pseudanabaena galeata* CCNP1313 against breast cancer cells, encouraged us to further investigate the anticancer potential of metabolites produced by the strain. Unlike previous studies that focused primarily on cellular extracts, we aimed to assess the cytotoxicity of chromatographic fractions using a broad panel of cancer cells, including those most commonly diagnosed (lung, breast, prostate, and cervical cancer). Additionally, we sought to identify the metabolites responsible for the observed effects through bioactivity-based molecular networking.

## 2. Results

The flash chromatography step gradient yielded a total of twenty-six fractions designated as Fx.y, where x represents the MeOH concentration in eluting solvent and y indicates the fraction number eluted with the solvent (for example, F20%.2 stands for second fraction eluted with 20% MeOH).

In the first step of the study, the cytotoxicity of *P. galeata* CCNP1313 cellular extract (200 µg mL^−1^) against A549, C33-A, CaSki, DoTC2, HDFa, HeLa, PC3, SiHa, and T47D cells was tested. After 24 h exposure, the sample demonstrated high cytotoxicity against all cancer cells except for the A549 and DoTC2 cells (Table 1). The extract reduced the relative viability of C33-A, CaSki, HeLa, PC3, SiHa, and T47D cells by approximately 90% (Table 1). It had a similar effect on healthy cells, reducing the viability of HDFa by 92% (Table 1).

Then, the activities of twenty-six flash chromatography fractions (200 µg mL^−1^) were tested against the cancer cell lines identified as sensitive to the cellular extract (C-33A, CaSki, HeLa, PC3, SiHa, and T47D) and HDFa cells. In the assay, T47D, C-33A, and CaSki cells were affected by the highest number of fractions (F70%.3–100%.4, F80%.2–100%.4, and F80%.2–100%.4, respectively; Table 2). These cells also exhibited the strongest cytotoxic responses, with a reduction in the relative cell viability by 89–95% (Table 2). In contrast, fraction F100%.4 was the only fraction active against PC3 cells; however, the observed effects were mild (45% reduction of relative cell viability; Table 2). While most of the fractions from F20%.1 to F70%.1 were inactive, F20%.1 was an exception, as it significantly reduced the viability of HeLa, HDFa, and SiHa cells by 70, 61, and 53%, respectively (Table 2).

When tested over a concentration range (25–200 µg mL^−1^), the samples reduced the relative cell viability in a dose-dependent manner (Figures S1–S7). To quantify the effects, IC_50_ values were calculated. The most potent cytotoxicity against cancer cells was observed for fraction F20%.1 against HeLa cells (IC_50_ = 83 ± 7 µg mL^−1^; Table S1, Figure S8), fractions F100%2 and F100%.4 against CaSki cells (IC_50_ = 84 ± 6, and 92 ± 11 µg mL^−1^, respectively; Table S1, Figure S8), and fractions F90%.2, F100%.3, and F100%.4 against C-33A cells (IC_50_ = 89 ± 6, 95 ± 13, and 94 ± 5 µg mL^−1^, respectively; Table S1, Figure S8). Notably, the overall strongest cytotoxicity was observed for F20%.1 against HDFa cells (IC_50_ = 66 ± 5 µg mL^−1^; Table S1, Figure S8).

The contents of the flash chromatography fractions were characterised with the application of a high-resolution mass spectrometry (HRMS) system. In the samples, eight previously identified galeapeptins (GP598, GP655, GP15, GP725, GP729, GP818, GP828, and GP999) and one previously identified *Pseudanabaena galeata* peptide (PG638) were detected (Table 3) [16]. The peptides were present in fractions F60%.1–F100%.4 but were absent in samples from the F20%–F50% range. The highest precursor ion intensities of the peptides were observed in fractions F60%.1–F60%.3 (Table 3). PG638 was detected exclusively in fraction F60%, whereas galeapeptins were more widely distributed, with GP729 present in each of the F60%.1–F100%.4 fractions (Table 3).

To gain better insights into the compounds responsible for the cytotoxicity, statistical analyses based on the method described by Nothias et al. [22] were performed. For each cancer cell line, a score for the top thirty most probable active features, defined as Spearman’s ρ × log_10_(max intensity), was computed. Heatmaps of these scores revealed that some of the features, identified as being potentially the most cytotoxic, were shared across multiple cell lines (Figures S9–S15, Table 4). Two features (*m*/*z* 563.4675 and 763.4997) were associated with activity against all cancer cell lines except HeLa; at the same time the feature at *m*/*z* 763.4997 was not selected as being active against HDFa cells (Table 4). Nine of the features were linked to activity against four cancer cells and, with the exception of feature at *m*/*z* 589.4825, they were all active against C-33A, CaSki, SiHa, and T47D cells (Table 4). Notably, forty-seven features were designated as being potentially responsible for the activity against only one cancer cell line, primarily HeLa, PC3, or SiHa; at the same time only several of those were also marked as cytotoxic to HDFa cells (Table 4).

Next, the relationship between cytotoxicity and fraction composition was analysed using CaSki cells, as this cell line was one of the three exhibiting the most pronounced responses. The statistical outputs (R) were mapped onto a molecular network generated in GNPS and visualised in Cytoscape 3.10.3 (Figure S16). The Bonferroni multiple testing correction identified thirty-two features as significant (adjusted *p* values < 0.05; Table 5). Based on the structures they were assigned to one of three lipid classes. The features whose ESI^+^ MS/MS spectra were dominated by fragments directly derived from acyl chain(s), including characteristic acylium ions [RCO]^+^ and related alkyl/alkenyl fragments, were classified as fatty acids (FAs). If, in addition to these criteria, they exhibited neutral losses corresponding to individual acyl chains (the loss of RCOOH or the corresponding ketene; Δm consistent with the mass of the relevant FA), yielding monoacylglycerol (MAG) ions, and showed products of glycerol backbone fragmentation and complementary ion pairs following the loss of a single acyl chain, such as [M + H − R_1_COOH]^+^ and [M + H − R_2_COOH]^+^, they were classified as diacylglycerols (DAGs). Lastly, features were classified as glycolipids, mono-, and digalactosyldiacylglycerols (MGDGs, DGDGs) when they met the DAG criteria and also showed evidence of a sugar headgroup alongside a diacylglycerol core, which was confirmed by a neutral loss of the hexose unit (Δm ≈ 162.0528 Da) from the precursor. DGDG was further distinguished by the sequential loss of two hexose units (2 × 162 Da), accompanied by the presence of co-occurring DAG/MAG-like ions after the cleavage of the glycan moiety and single acyl chain.

**Figure 1:**
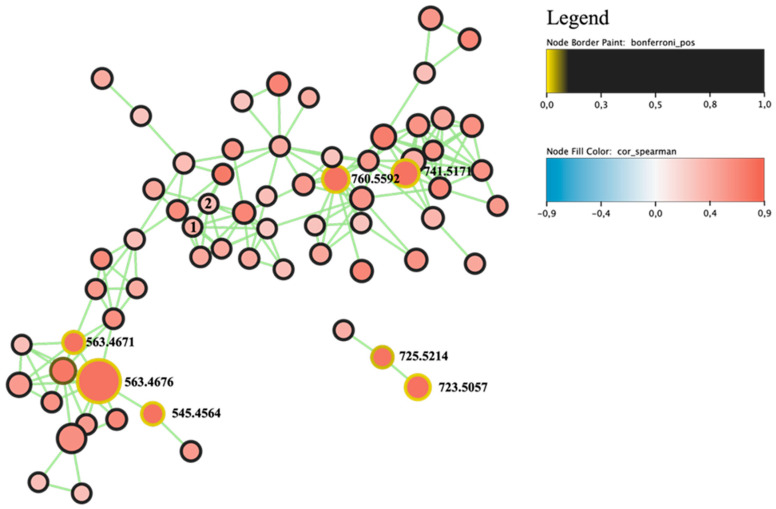
Cluster containing compounds most likely responsible for the cytotoxicity of *Pseudanabaena galeata* CCNP1313 against the CaSki cell line. Node size corresponds to the precursor ion intensity, node colour represents the Spearman correlation (blue—negative, white—zero, red—strong positive), and the node border indicates significance after Bonferroni’s multiple testing correction (yellow—positive, dark—negative). For the selected features, the precursor *m*/*z* values were labelled. Features annotated in GNPS were marked as 1 and 2.

The molecular network workflow revealed two clusters that represent the most likely groups of bioactive compounds (Figure 1). The first, larger cluster comprised sixty-three nodes connected by one hundred sixty-one edges, five of which were prioritised by Bonferroni’s correction. The second, smaller cluster (three nodes, two edges), contained two significant nodes and was highly structurally similarity to the first. The remaining twenty-five significant features were either isolated nodes or part of larger, poorly correlated clusters.

**Figure 2:**
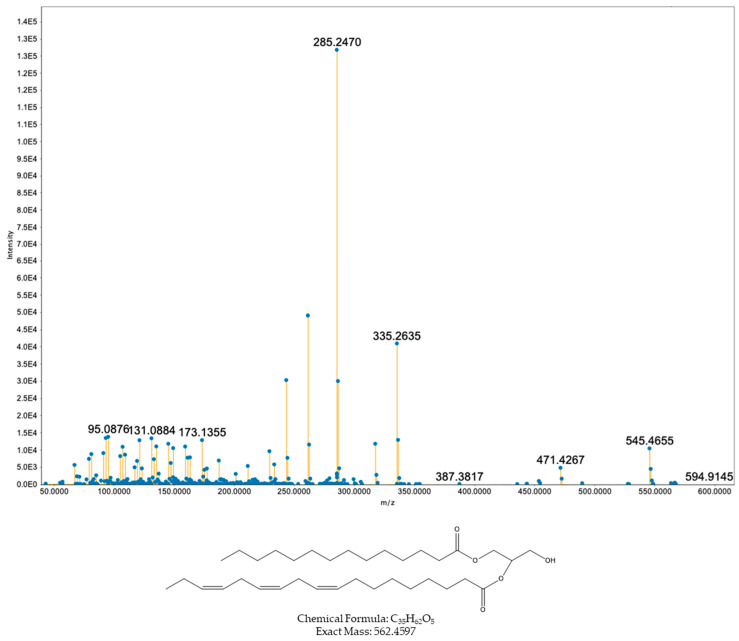
MS/MS spectrum and proposed structure of compound with precursor ion *m*/*z* 563.4676 ([M + H]^+^) assigned as a diacylglycerol DAG (18:3/14:0; double-bond position not assigned).

The first three nodes at *m*/*z* values 563.4671, 563.4676, and 545.4564 were assigned to the diacylglycerol species DAG (18:3/14:0) (Figure 2). This assignment is supported by the excellent agreement between the measured and calculated exact masses for a DAG bearing 18:3 and 14:0 acyl chains (mass error 0.00007 Da), and also by the diagnostic product ions. Complementary fragments are generated by neutral losses matching the free fatty acids 18:3 and 14:0 (Δm ≈ 278.2246 Da and Δm ≈ 228.2089 Da), yielding ions at ~*m*/*z* 285.24 and ~*m*/*z* 335.26 that are characteristic of DAG cleavages. In addition, a loss of the glycerol backbone was present (Δm ≈ 92.0473 Da) to give ~*m*/*z* 471.42.

**Figure 3:**
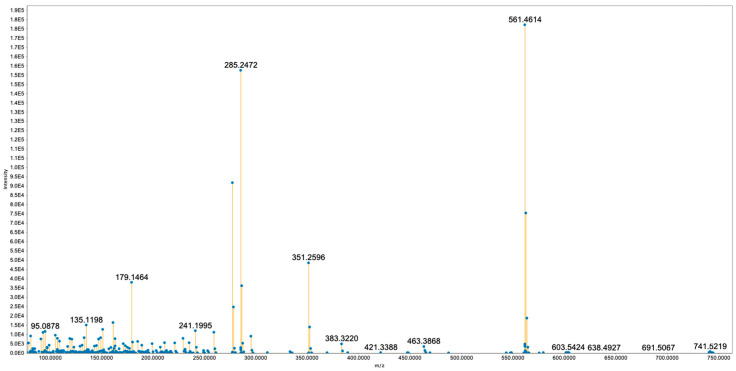
Spectrum of compound with precursor ion *m*/*z* 741.5219 ([M + H]^+^) assigned as the monogalactosyldiacylglycerol MGDG (18:3/14:0).

The remaining nodes at *m*/*z* values 760.5592 and 741.5171 were assigned to MGDGs (Figure 3). Their spectra display the expected DAG core series after glycosidic cleavage (Δm ≈ 162.0528 Da) together with a very intense ion at *m*/*z* ~285.247 consistent with a C18:3-derived fragment. However, the other peaks do not unambiguously support a specific identity for the second acyl chain, which prevents the precise structural annotation of these two features.

**Figure 4:**
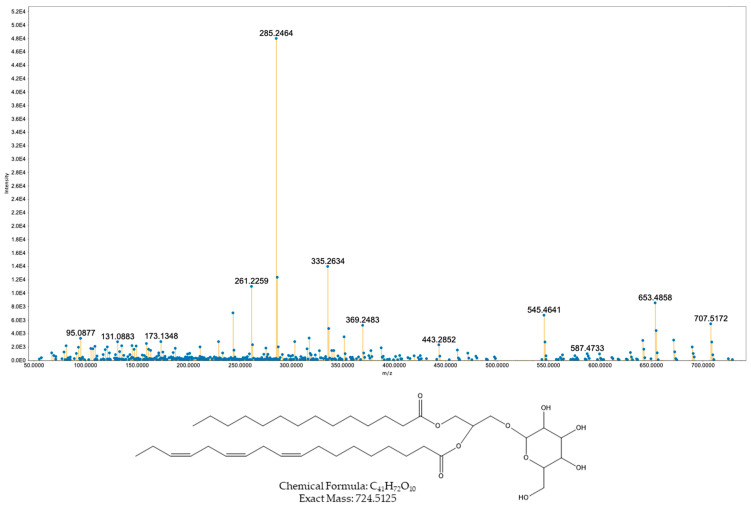
MS/MS spectrum and proposed structure of the compound with precursor ion *m*/*z* 725.5214 ([M + H]^+^) assigned as a diacylglycerol, DAG (18:3/14:0; double-bond positions not assigned).

**Figure 5:**
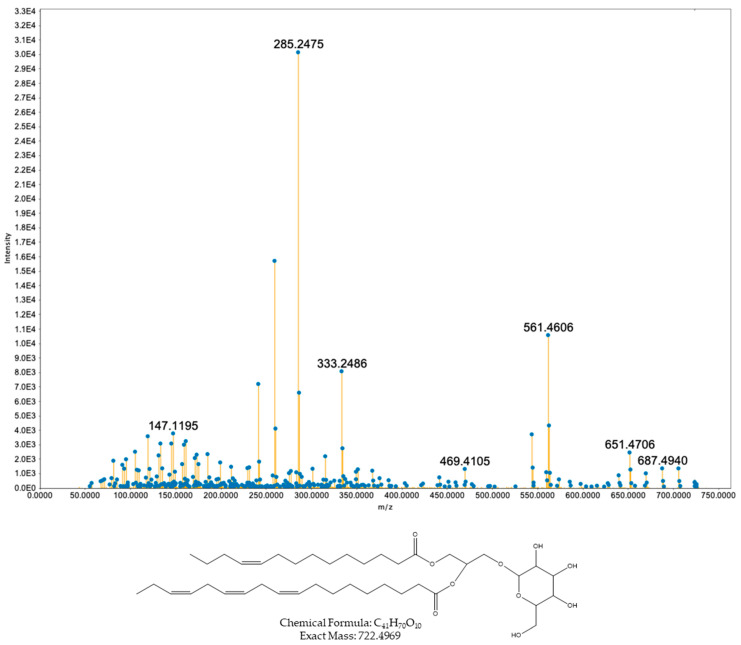
MS/MS spectrum and proposed structure of the compound with precursor ion *m*/*z* 723.5057 ([M + H]^+^) assigned as a diacylglycerol, DAG (18:3/14:1; double-bond positions not assigned).

Features in the second cluster at *m*/*z* values 725.5214 and 723.5057 were assigned to MGDG (18:3/14:0) and MGDG (18:3/14:1), with mass errors of 0.0011 and 0.0010, respectively (Figure 4 and Figure 5). Both spectra show the expected glycosidic cleavage and the associated DAG core fragment series.

## 3. Discussion

Works published within the last decade have demonstrated that Baltic Sea cyanobacteria possess significant biotechnological and pharmaceutical potential [16,20,23,24,25,26,27,28,29,30,31,32,33,34]. In the first study on the Baltic strain CCNP1313, ethanol extract was active against MCF-7 (IC_50_ = 100 µg mL^−1^), without affecting HDFa cells. Additionally, the cytotoxic effects were determined to result from apoptosis rather than necrosis [20]. In a subsequent study, flash chromatography fractions from the same strain were highly cytotoxic to the T47D breast cancer cells, reducing its viability by more than 90%. Some of the fractions also affected HDFa cells; however, the observed effects were less pronounced (60% reduction) [16]. In the current work, the cytotoxicity of CCNP1313 was tested against a wider panel of cancer cells (A549, C-33A, CaSki, DoTC2, HeLa, PC3, SiHa, and T47D). The obtained results confirmed the activity of CCNP1313 against T47D cells and showed high cytotoxicity of the extract against four out of five cervical cancer cell lines (C-33A, CaSki, HeLa, and SiHa) and PC3 cells. Further studies were conducted with the application of the sensitive cells (C-33A, CaSki, HeLa, PC3, SiHa, and T47D) and chromatographically separated fractions. Consistent with earlier results [16], the selective cytotoxicity of hydrophobic fractions against T47D cells was observed (Table 2). The susceptibility of T47D cells to metabolites produced by *Pseudanabaena* has also been reported for strains isolated from Portugal [19]. The extracts additionally reduced the viability of another breast cancer cell line (SK-BR-3) [19]. While this does not indicate that the same compounds are responsible for the activity of both the Baltic and Portuguese strains, it cannot be excluded that they belong to the same class of cyanometabolites. Our study, also revealed that cervical cancer cells, primarily C-33A and CaSki, were the most sensitive to CCNP1313 fractions, while the highest selectivity was observed against PC3 cells (Table 2).

The tested fractions inhibited cancer cell growth in a dose-dependent manner (Figures S1–S7), with IC_50_ values against T47D cells ranging from 107 ± 18 to 200 ± 30 µg mL^−1^. The direct comparison of these findings with similar studies is challenging, as IC_50_ values are rarely reported or other cancer cell lines were used. In a few studies reporting IC_50_ values, the cytotoxicity of cyanobacterial material was evaluated against MCF7 or T47D cells using *Spirulina subsalsa* [27], *Nostoc linckia* [35,36], *Geitlerinema carotinosum*, *Chroococcus minutus*, *Anabaena oryzae* [35], and *Arthrospira platensis* [37]. The resulting IC_50_ values ranged from 27.99 to 189.45 µg mL^−1^. Additionally, activity against cervical cancer cells HeLa or SiHa has also been assessed, with the strongest effects (IC_50_ = 26.80 µg mL^−1^) induced by *Fischerella major* [37,38,39,40]. In the present study, the selectivity index for fraction F20%.1 indicates stronger cytotoxicity towards HDFa cells than towards HeLa and SiHa cells. However, it cannot be ruled out that this fraction contains more than one biologically active agent and that each of them acts on a different cell type. Therefore, the calculated IC_50_ values for these type of samples (i.e., extracts and fractions) do not reflect the true potency of the active components, as at this stage their concentrations are unknown. Only assays performed on the isolated pure metabolites can provide reliable information on the biological activity of natural products.

Our works have consistently demonstrated the cytotoxicity of CCNP1313 against breast and cervical cancer cells. However, identifying the active metabolites remains a significant challenge, likely because they might be synthesised in minute quantities. This was especially evident in the case of dolastatin 10, for which nearly two decades passed between the discovery of the potent cytotoxicity of the biological material and identifying the bioactive molecule [41]. Despite its adverse effects, dolastatin derivatives became the foundation for the successful development of antibody–drug conjugates (ADCs) as approved drugs (Brentuximab vedotin, Polatuzumab vedotin, Enfortumab Vedotin-ejfv, Disitamab Vedotin, Tisotumab vedotin-tftv, Telisotuzumab vedotin, and Belantamab Mafodotin-blmf) effective in the treatment of breast, cervical, ovarian, urothelial, lung, and gastric cancers, as well as lymphoma, soft tissue sarcoma, myeloma, and leukaemia (https://www.marinepharmacology.org/approved (accessed on 1 October 2025).

The success story of dolastatin, combined with the genomic data suggesting that *P. galeata* has the potential to synthesise bioactive compounds [2], motivates our search for molecules responsible for the observed cytotoxicity. In recent years these kinds of studies have been accelerated through the application of advanced bioinformatics tools. Here, in order to establish the relationship between the chemical composition of CCNP1313 chromatographic fractions and their cytotoxicity, molecular networking was employed. These analyses resulted in the identification of glycoglycerolipids, DAGs (*m*/*z* values 563.4671, 563.4676, and 545.4564), and MGDGs (*m*/*z* values 760.5592, 741.5171, 725.5214, and 723.5057) as the most plausible sources of bioactivity. Glycoglycerolipids constitute a major class of lipids in photosynthetic organisms such as cyanobacteria (including *Pseudanabaena* sp. M2) [42,43,44,45,46,47], algae [48,49,50,51], and higher plants [52,53,54]. The compounds play a fundamental role in cyanobacterial cells by forming the core lipid matrix of thylakoid membranes and ensuring the stability and efficient functioning of the photosynthetic apparatus [52]. Environmental abiotic stress factors, such as nutrient deficiency, high light intensity, or the absence of light, affect the lipid profile and their accumulation in microalgal cells [55,56,57,58,59,60]. In our efforts to enhance the biomass production of CCNP1313, we optimised the culture parameters of the strain, including growth media, light intensity, and biomass harvesting protocol. The cells are collected in the mid-exponential phase of growth prior to nutrient depletion to ensure optimal physiological conditions and maximise biomass yield. In numerous studies, the activity of glycoglycerolipids was explored [61]. A glycolipid fraction from spinach (*Spinacia oleracea* L.) containing MGDG, DGDG, and sulfoquinovosyl diacylglycerol (SQDG) [62], as well as purified MGDG [63], inhibited the proliferation of HeLa, A549, BALL-1 (acute lymphoblastoid leukaemia), HCT116 (colon cancer), HL60 (promyelocytic leukaemia), and NUGC-3 (stomach cancer) cells with IC_50_ values ranging from 39.20 to 57.20 µg mL^–1^. MGDG isolated from a single-celled cyanobacterium of the genera *Synechocystis* induced apoptosis in breast cancer cell lines (BT-474, MDA-MB-231) at IC_50_ = 27.20 and 150.00 ng mL^−1^ [47]. Additionally, MGM also demonstrated synergistic effects with gemcitabine, a commonly used anticancer drug, inhibiting the proliferation of human pancreatic cancer cells (BxPC-3, MiaPaCa-2, and PANC-1) [64]. Additionally, it has been demonstrated that certain lipids can interact with proteins to form complexes that induce apoptosis across a broad spectrum of cancer cell types without affecting healthy cells [65,66,67,68,69,70]. When tested independently, the effects of the protein were not as strong as those of the complex [69,71].

Published studies on MGDGs and DGDGs activity strongly support the results of the molecular networking performed in the current work. Molecular networking is an increasingly and successfully employed approach for identifying natural products responsible for biological activity. For example, based on this method the antiviral effects of chromatographic fractions from *Euphorbia dendroides* L. (Euphorbiaceae) latex (a Mediterranean tree spurge) were attributed to 4*β*-deoxyphorbol esters. The compounds were subsequently isolated and their activity was confirmed with in vitro methods [72]. Bioinformatics tools not only facilitate the investigation of natural products responsible for the observed bioactivity, but also significantly reduce the time and costs of the studies.

## 4. Conclusions

In this study, as in many investigations of microalgae, obtaining a sufficient quality of biomass for the identification of bioactive compounds is a major limiting factor. For *Pseudanabaena galeata* CCNP1313, approximately one year was required to collect enough material for cytotoxicity assessment, whereas even greater amounts are necessary for metabolite isolation and structural identification. Molecular networking substantially reduced the time and effort required to identify the metabolites responsible for the observed biological activity. This approach enabled the identification of lipids as the most probable cytotoxic constituents synthesised by CCNP1313. The definitive confirmation of their role in cytotoxicity will depend on the isolation of these compounds and the validation of their activity through targeted bioassays.

## 5. Materials and Methods

### 5.1. Culture Conditions, Extraction, and Fractionation of Pseudanabaena galeata CCNP1313 Metabolites

CCNP1313 (GenBank accession number MN273769) was cultured in BG11 medium [16]. Cells were harvested by centrifugation (4000× *g*; 15 min; 4 °C) and lyophilised. In the first step of the study, 2 g of freeze-dried material were extracted with 75% methanol (MeOH) in MilliQ water (2 × 50 mL) by vortexing (20 min) The obtained samples were combined and centrifuged (4000× *g*; 15 min; 4 °C).

To further assess the potential of the strain, in the second step of the study, 20 g of freeze-dried CCNP1313 cells were extracted using 75% MeOH (2 × 250 mL) by vortexing (30 min). After centrifugation, the supernatant was diluted with MilliQ water to a MeOH concentration < 10% and fractionated on a Shimadzu HPLC system (Shimadzu Corporation, Kyoto, Japan) in a 120 g SNAP KP-C_18_-HS column (Biotage, Uppsala, Sweden). A step gradient of increasing MeOH concentration (20–100%) in MilliQ water was used.

The initial extract and the 40 mL chromatographic fractions were placed in pre-weighed vials, concentrated in a centrifugal vacuum concentrator (MiVac, SP Scientific, Ipswich, UK), and freeze-dried.

### 5.2. Cell Culture and Cytotoxicity Assessment

The cytotoxicities of the extract and chromatographic fractions were tested against a panel of cancer cell lines, including lung (A549), prostate (PC3), breast (T47D) (ECACC; Merck KGaA, Darmstadt, Germany), and cervical (HeLa; ECACC; C33-A, DoTC2, SiHa, CaSki; ATCC, Manassas, VA, USA). Human dermal fibroblasts (HDFa; Cell Applications; Merck) were used as a control. A549 cells were cultured in F-12K medium, T47D and CaSki cells were cultured in RPMI medium, C-33A, PC3, HeLa, and HDFa cells were cultured in DMEM medium, and SiHa cells were cultured in IMEM medium. All cells were incubated at 37 °C (±1 °C) and 5% CO_2_ in media supplemented with 10% foetal bovine serum and a penicillin–streptomycin solution (100 U and 100 mg mL^−1^, respectively). The media and supplements were from Gibco (Thermo Fisher Scientific Inc., Waltham, MA, USA).

For cytotoxicity assessment, the MTT (3-(4,5-dimethylthiazol-2-yl)-2,5-diphenyltetrazolium-bromide) assay was employed. First, samples were tested against all cell lines (200 µg mL^−1^), and in a further assay only active samples were used (25–200 µg mL^−1^). Cells were seeded in 96-well plates in their respective media at 4 × 10^3^ (A549, C33-A, CaSki, DoTC2, HeLa, PC3, SiHa, and T47D) and 10 × 10^3^ (HDFa) per well and incubated overnight (24 h; 37 °C; 5% CO_2_). The following day, cells were treated with samples and incubated (24 h; 37 °C; 5% CO_2_). Then the MTT solution (4 mg mL^−1^; Sigma-Aldrich, St. Louis, MO, USA) was added. After 4 h of incubation, formazan was dissolved with 100% DMSO, and absorbance was measured (Varioskan plate reader; Flash Thermo Fisher Scientific, Vantaa, Finland). In the tests, 1% DMSO served as a solvent control and 3,4-dichloroaniline (Sigma-Aldrich) as a positive control. Cell viability was calculated as the percentage of the mean absorbance of the sample relative to the mean absorbance of the solvent control. Cytotoxicity of the samples was determined in triplicate across three biologically independent experiments. Reduction in relative cell viability greater than 50% was considered significant. Statistical significance was evaluated using one-way ANOVA, with a Student’s *t*-test and Bonferroni’s correction. The significance level was established at *p* < 0.05. The IC_50_ values were calculated using log transformed dose–response curves.

### 5.3. High-Resolution Mass Spectrometry Analyses

The contents of flash chromatography fractions were analysed using a Waters Synapt XS high-resolution mass spectrometer (HRMS) coupled with an ACQUITY Premier UPLC system (Milford, MA, USA). The analytes were separated in an Acquity Premier BEH C_18_ column (2.1 mm × 50 mm; 1.75 µm; Waters), using a mixture of 5% acetonitrile in MilliQ water (phase A) and acetonitrile (phase B), both with formic acid (0.1%). The gradient was run at 0.4 mL min^−1^ from 1% to 30% B over 5 min, and then to 100% B over the subsequent 10 min. To minimise analytical bias, the sample injection order was randomised prior to analyses. Data were acquired with the MassLynx 4.2 SCN1028 software (Waters) using positive electrospray ionisation (ESI^+^). The ion source operated with a capillary voltage of 3.0 kV and a sampling cone voltage of 4 V. The source and desolvation temperatures were maintained at 40 °C and 490 °C, respectively. Cone and desolvation gas flow rates were 50 L h^−1^ and 1000 L h^−1^, with a nebuliser pressure of 5.2 bar. Full-scan mass spectra (*m*/*z* 100–2000) were recorded using fast data-dependent acquisition (fastDDA) with a scan time of 0.1 s. MS/MS fragmentation was performed with collision energies of 20–50 V for low-mass and 40–100 V for high-mass precursor ions. The instrument was calibrated using sodium formate, while leucine enkephalin ([M + H]^+^, *m*/*z* 556.2771) was used as a lock mass to ensure mass accuracy.

### 5.4. Mass Spectrometry Data Processing

High-resolution mass spectrometry data were first converted to the open-source .mzXML format using Waters Data Convert and subsequently imported to MZmine 4.6.1 [73] for processing. During this step, data were first filtered to remove all MS/MS fragment ions within ±17 Da of the precursor *m*/*z*, then the MS/MS spectra were filtered to retain only the top 6 fragment ions in the ±50 Da window throughout the spectrum. For the analyses, both the precursor ion mass and the MS/MS fragment ion tolerances were set to 0.02 Da. Then a molecular network was generated, using the Feature-Based Molecular Networking (FBMN) workflow [74] and the GNPS platform (Global Natural Products Social Molecular Networking; https://gnps.ucsd.edu (accessed on 19 September 2025). The edges in the network were filtered to have a cosine score greater than 0.6 and more than 6 matched peaks, and were only retained if each of the nodes appeared in the top 10 most similar nodes of the other. Then, the molecular families were limited to a maximum of 100 nodes by removing the lowest-scoring edges. Finally, the network spectra were searched against GNPS spectral libraries, which were filtered in the same manner as the input data. A match was considered valid only if it had a cosine score greater than 0.7 and at least 6 matched peaks.

### 5.5. Bioactivity-Based Molecular Networking Analyses

Bioactivity-based molecular networking analyses were performed using a modified workflow from Nothias et al. [72]. Briefly, cell viability data were first converted to inhibition values (100—viability) and then merged with LC–MS feature quantification tables from MZmine to allow direct correlation with metabolite intensities. For each feature, Pearson’s and Spearman’s correlation coefficients between bioactivity and log_10_-transformed peak intensities were calculated. To account for the relevance of the feature, an intensity-weighted score was also defined as Spearman’s rho × log_10_(max intensity). Subsequently, multiple testing correction was performed using both the Benjamini–Hochberg FDR and Bonferroni’s procedures. However, this analysis was restricted to features that demonstrated a positive correlation (Text S1).

### 5.6. Data Visualisation

Heatmaps of the top 30 ranked features were generated with the ComplexHeatmap R package 2.13.1. The heatmaps display metabolite intensities on a log_10_ scale (white to red) and annotate each fraction with its corresponding cell viability value, allowing for a direct comparison between chemical signal distribution and biological activity profiles. Molecular networks were visualised in Cytoscape 3.10.3 software [75] using a consistent visual style across all figures. Node sizes were assigned to the precursor signal (log_10_ intensity), the colour of the nodes represents Spearman’s ρ (using a diverging blue–white–red palette for negative–zero–positive association), and the borders of the nodes indicate multiple testing significance (e.g., solid border for Bonferroni-significant features). To identify the source of activity, the nodes on the GNPS map were ranked by CaSki-specific intensity-weighted score and Bonferroni’s significance, which highlighted a single, spectrally coherent subnetwork as the most plausible source of activity.

## Figures and Tables

**Table 1:** Relative cell viability of cancer cells (A549, C-33A, CaSki, DoTC2, HeLa, PC3, SiHa, and T47D) and human dermal fibroblasts (HDFas) exposed to *Pseudanabaena galeata* CCNP1313 cellular extract applied at 200 µg mL^−1^ (data presented as mean values with standard deviation). The statistical analyses were performed using a one-way ANOVA with significance against the control indicated by an asterisk for *p* < 0.05.

Relative Cell Viability [%]								
A549	C-33A	CaSki	DoTC2	HDFa	HeLa	PC3	SiHa	T47D
121 ± 7 *	8 ± 4 *	10 ± 3 *	80 ± 7 *	8 ± 2 *	14 ± 5 *	13 ± 3 *	3 ± 2 *	8 ± 3 *

**Table 2:** Relative cell viability of cancer cells (C-33A, CaSki, HeLa, PC3, SiHa, and T47D) and human dermal fibroblasts (HDFa) exposed to the *Pseudanabaena galeata* CCNP1313 chromatographic fractions at 200 µg mL^−1^ (data presented as mean values; active fractions are marked in orange). The fractions are designated as Fx.y, where x represents the MeOH concentration in eluting solvent and y indicates the fraction number eluted with the solvent (for example, F20%.2 stands for second fraction eluted with 20% MeOH). The statistical analyses were performed using a one-way ANOVA with significance against the control indicated by an asterisk for *p* < 0.05.

Sample	Relative Cell Viability [%]						
	C-33A	CaSki	HDFa	HeLa	PC3	SiHa	T47D
**F20%.1**	72 *	66 *	39 *	30 *	63 *	7 *	59 *
**F20%.2**	81 *	65 *	102	59 *	83 *	97	87 *
**F30%.1**	60 *	98	100	74 *	90 *	121 *	99
**F30%.2**	91 *	86 *	109 *	65 *	89 *	114 *	94 *
**F40%.1**	86 *	92 *	103	63 *	88 *	114 *	95 *
**F40%.2**	86 *	90 *	112 *	71 *	89 *	113 *	89 *
**F50%.1**	78 *	90 *	87 *	61 *	83 *	120 *	93 *
**F50%.2**	89 *	93 *	110 *	72 *	101	121 *	98
**F50%.3**	89 *	98 *	111 *	71 *	100	121 *	100
**F60%.1**	84 *	102	115 *	79 *	98	111 *	94 *
**F60%.2**	85 *	101	111 *	71 *	94 *	116 *	81 *
**F60%.3**	73 *	96	113 *	80 *	99	122 *	80 *
**F70%.1**	70 *	92 *	109 *	86 *	95 *	115 *	60 *
**F70%.2**	53 *	80 *	95 *	53 *	78 *	94 *	51 *
**F70%.3**	63 *	90 *	112 *	78 *	90 *	119 *	50 *
**F80%.1**	53 *	81 *	107 *	85 *	92 *	117 *	44 *
**F80%.2**	33 *	48 *	123 *	75 *	87 *	94 *	40 *
**F80%.3**	28 *	41 *	116 *	76 *	87 *	73 *	42 *
**F80%.4**	28 *	37 *	106 *	76 *	80 *	53 *	42 *
**F90%.1**	22 *	22 *	114 *	66 *	88 *	79 *	41 *
**F90%.2**	27 *	34 *	60 *	68 *	80 *	61 *	34 *
**F90%.3**	18 *	16 *	108 *	70 *	79 *	38 *	27 *
**F100%.1**	32 *	37 *	91 *	85 *	85 *	100	34 *
**F100%.2**	17 *	9 *	51 *	48 *	60 *	32 *	12 *
**F100%.3**	15 *	8 *	54 *	55 *	64 *	19 *	12 *
**F100%.4**	7 *	5 *	56 *	29 *	45 *	19 *	11 *

**Table 3:** Heatmap of galeatapeptins (GPs) and *Pseudanabaena galeata* peptides (PGs) detected in chromatographic fractions from *Pseudanabaena galeata* CCNP1313. Colour intensity (light to dark orange) represents the relative abundance of each of the peptides, calculated from the peak area of its precursor ion, and n.d. stands for not detected.

Compound	*m*/*z*	Fraction																	
		F20%–F50%	F60%			F70%			F80%				F90%			F100%			
			1	2	3	1	2	3	1	2	3	4	1	2	3	1	2	3	4
**GP598**	**599.4133**	n.d.	1.37 × 10^4^	5.82 × 10^1^	9.54 × 10^2^	1.32 × 10^3^	n.d.	n.d.	n.d.	n.d.	n.d.	4.24 × 10^3^	n.d.	n.d.	n.d.	n.d.	n.d.	n.d.	n.d.
**PG638**	**639.3181**	n.d.	1.43 × 10^4^	n.d.	4.32 × 10^3^	n.d.	n.d.	n.d.	n.d.	n.d.	n.d.	n.d.	n.d.	n.d.	n.d.	n.d.	n.d.	n.d.	n.d.
**GP655**	**656.8993**	n.d.	1.53 × 10^4^	n.d.	1.42 × 10^2^	n.d.	n.d.	n.d.	n.d.	n.d.	n.d.	1.01 × 10^3^	n.d.	n.d.	n.d.	n.d.	n.d.	n.d.	n.d.
**GP715**	**716.4362**	n.d.	6.28 × 10^4^	n.d.	8.06 × 10^4^	n.d.	n.d.	2.48 × 10^2^	n.d.	2.81 × 10^3^	2.48 × 10^2^	4.87 × 10^3^	1.29 × 10^3^	n.d.	n.d.	n.d.	n.d.	n.d.	n.d.
**GP725**	**726.3513**	n.d.	1.37 × 10^4^	n.d.	nd	n.d.	n.d.	n.d.	n.d.	n.d.	n.d.	n.d.	n.d.	n.d.	n.d.	n.d.	n.d.	n.d.	n.d.
**GP729**	**730.4509**	n.d.	8.44 × 10^4^	5.91 × 10^2^	1.03 × 10^5^	1.10 × 10^3^	4.22 × 10^3^	3.68 × 10^2^	3.10 × 10^3^	2.29 × 10^3^	3.73 × 10^2^	6.42 × 10^3^	1.68 × 10^3^	2.88 × 10^3^	2.56 × 10^3^	1.00 × 10^3^	1.35 × 10^3^	1.31 × 10^3^	2.16 × 10^2^
**GP818**	**819.5004**	n.d.	n.d.	n.d.	7.02 × 10^3^	n.d.	n.d.	n.d.	n.d.	n.d.	n.d.	n.d.	n.d.	n.d.	n.d.	n.d.	n.d.	n.d.	n.d.
**GP828**	**829.5208**	n.d.	n.d.	n.d.	n.d.	n.d.	5.66 × 10^2^	n.d.	2.75 × 10^3^	2.43 × 10^3^	n.d.	1.30 × 10^3^	n.d.	n.d.	n.d.	n.d.	n.d.	n.d.	n.d.
**GP998**	**999.6283**	n.d.	n.d.	n.d.	6.55 × 10^3^	n.d.	n.d.	3.16 × 10^3^	3.41 × 10^4^	n.d.	2.87 × 10^2^	7.01 × 10^1^	n.d.	n.d.	9.63 × 10^2^	n.d.	n.d.	n.d.	n.d.

**Table 4:** Top LC-MS features correlated with cytotoxic activity of *Pseudanabaena galeata* CCNP1313 against cancer cells (C-33A, CaSki, HeLa, PC3, SiHa, and T47D) and human dermal fibroblasts (HDFa). Features denoted as active only against HDFa cells were excluded. Compounds with identical or highly similar MS spectra were grouped together, with mass difference indicated in the brackets. A “+” indicates features identified as being responsible for the observed cytotoxicity.

*m*/*z*	Activity Against						
	C-33A	CaSki	HDFa	HeLa	PC3	SiHa	T47D
207.1361				+			
219.1467					+		
221.1517	+						+
225.1466					+		
225.1569				+			
251.1622	+	+				+	+
257.1269				+			
265.1417				+			
277.2144 (±0.0001)	+						
279.2300	+	+				+	+
282.2772	+	+				+	+
285.2408 (±0.0004)			+	+			
289.1780	+	+					
303.1321				+			
309.4792					+		
322.1996					+		
333.2155					+		
335.2178	+	+				+	+
335.2565	+	+					+
337.2711 (±0.0004)			+		+	+	
340.2575					+		
350.1525	+	+				+	
353.2172	+						
353.2287 (±0.0002)	+	+				+	+
391.223	+	+					+
394.1432	+		+			+	+
405.1667				+			
407.2168					+		
425.2275					+		
455.1573	+				+		
531.3165				+			
542.3255				+			
545.4564	+	+					+
547.4725 (±0.0005)			+	+	+		
553.2986	+	+				+	+
556.3051					+		
561.4517	+	+					+
563.4675 (±0.0007)	+	+	+		+	+	+
563.4671	+	+				+	+
569.2938	+						+
573.2365				+			
575.2547	+						
577.2682						+	
579.2844	+	+					+
579.4617						+	
580.3329				+			
589.3192				+			
589.4825 (±0.0004)		+			+	+	+
611.3179				+			
633.3594				+			
704.5299			+		+		
707.5107	+	+					+
715.5839				+			
718.5453			+	+			
723.5057	+	+					+
725.3865				+			
725.5214	+	+					+
741.5170		+					
741.5171	+	+					+
744.5640				+			
746.5799				+			
747.5040				+			
760.5592		+			+	+	
763.4997 (±0.0004)	+	+			+	+	+
765.5152 (±0.0001)					+	+	
770.5802				+			
772.5962				+			
775.3880				+			
779.4948 (±0.0001)			+			+	
788.5908						+	
791.5309 (±0.0007)	+	+				+	+
801.5524				+			
807.5264					+	+	+
817.5467			+		+	+	
819.5604				+			
863.6228			+		+		
863.6238			+	+			
877.6397			+		+	+	
906.6187			+		+		
908.6340			+		+		
909.5584					+	+	
911.5744			+		+	+	

**Table 5:** Features significant for the activity of *Pseudanabaena galeata* CCNP1313 against CaSki cell line after Bonferroni’s multiple testing correction. FA stands for fatty acid, DAG stands for diacylglycerol, MGDGs stands for monogalactosyldiacylglycerols, PQMS stands for poor-quality mass spectrum, which made it impossible to assign the feature to any of the known compound classes.

Feature	*m*/*z*	Bonferroni	Retention Time	Sum Area
FA	279.22998	0.000	24.384	4.2 × 10^4^
MGDG	763.49993	0.000	32.736	1.6 × 10^4^
MAG	335.21721	0.001	20.581	2.0 × 10^4^
MGDG	763.49932	0.003	34.228	2.9 × 10^4^
PQMS	394.14324	0.003	25.013	9.3 × 10^3^
FA	251.16223	0.004	17.598	2.2 × 10^4^
PQMS	353.22855	0.009	18.025	1.6 × 10^4^
MAG	335.2178	0.010	22.025	5.1 × 10^4^
DAG	589.48208	0.013	33.961	4.9 × 10^3^
FA	282.27723	0.014	29.775	5.0 × 10^3^
DAG	553.29857	0.015	19.188	2.4 × 10^4^
MGDG	723.50568	0.016	32.744	1.0 × 10^4^
MGDG	791.53029	0.016	33.979	2.7 × 10^3^
PQMS	391.22303	0.017	33.681	4.5 × 10^3^
DAG	563.46758	0.018	33.681	3.3 × 10^4^
DAG	563.46711	0.018	31.362	3.3 × 10^3^
MAG	335.2565	0.018	33.681	2.8 × 10^3^
DAG	545.45635	0.018	33.681	4.7 × 10^3^
MGDG	707.51071	0.018	33.681	8.6 × 10^3^
MGDG	741.51706	0.018	34.038	7.9 × 10^3^
MGDG	760.55916	0.019	35.046	8.6 × 10^3^
DAG	561.45171	0.019	34.034	1.3 × 10^4^
FA	350.15251	0.021	24.390	8.1 × 10^3^
DAG	579.2844	0.022	24.155	1.3 × 10^4^
DGDG	909.55839	0.024	34.598	1.4 × 10^4^
MGDG	725.52141	0.027	33.704	4.8 × 10^3^
DAG	563.46753	0.029	34.951	8.0 × 10^3^
FA	289.17797	0.031	18.708	3.5 × 10^4^
DAG	579.28396	0.036	20.103	9.1 × 10^3^
DGDG	911.57439	0.039	35.657	1.7 × 10^4^
PQMS	455.1573	0.041	5.270	6.3 × 10^3^
MGDG	807.52642	0.048	33.090	5.1 × 10^3^

## Data Availability

Data obtained during this study is available either in the manuscript or Supplementary Materials. Further inquiries can be directed to the corresponding authors.

## Supplementary Materials

The following supporting information can be downloaded at: https://www.mdpi.com/article/10.3390/toxins17120586/s1, Text S1: Bioactivity-Based Molecular Networking Analyses; Table S1: Inhibitory effects on cancer (C-33A, CaSki, HeLa, PC3, SiHa, T47D) and healthy (HDFa) cells exposed to flash chromatography fractions from *Pseudanabaena galeata* CCNP1313. The fractions are designated as Fx.y, where x represents the MeOH concentration in eluting solvent and y indicates the fraction number eluted with the solvent (for example, F20%.2 stands for second fraction eluted with 20% MeOH). Data are presented as mean IC_50_ values (µg mL^−1^) with standard deviation, 95% confidence intervals, and selectivity index for fraction F20%.1.; Figure S1: Relative cell viability of C33A cells exposed to flash chromatography fractions from *Pseudanabaena galeata* CCNP1313 and 3,4-dichloroaniline (3,4-DCA) (data are presented as mean values with standard deviation). The fractions are designated as Fxy, where x represents the MeOH concentration in eluting solvent and y indicates the fraction number eluted with the solvent (for example, F20%2 stands for second fraction eluted with 20% MeOH); Figure S2: Relative cell viability of CaSki cells exposed to flash chromatography fractions from *Pseudanabaena galeata* CCNP1313 and 3,4-dichloroaniline (3,4-DCA) (data are presented as mean values with standard deviation). The fractions are designated as Fxy, where x represents the MeOH concentration in eluting solvent and y indicates the fraction number eluted with the solvent (for example, F20%2 stands for second fraction eluted with 20% MeOH); Figure S3: Relative cell viability of HDFa cells exposed to flash chromatography fractions from *Pseudanabaena galeata* CCNP1313 and 3,4-dichloroaniline (3,4-DCA) (data are presented as mean values with standard deviation). The fractions are designated as Fxy, where x represents the MeOH concentration in eluting solvent and y indicates the fraction number eluted with the solvent (for example, F20%2 stands for second fraction eluted with 20% MeOH); Figure S4: Relative cell viability of HeLa cells exposed to flash chromatography fractions from *Pseudanabaena galeata* CCNP1313 and 3,4-dichloroaniline (3,4-DCA) (data are presented as mean values with standard deviation). The fractions are designated as Fxy, where x represents the MeOH concentration in eluting solvent and y indicates the fraction number eluted with the solvent (for example, F20%2 stands for second fraction eluted with 20% MeOH); Figure S5: Relative cell viability of PC3 cells exposed to flash chromatography fractions from *Pseudanabaena galeata* CCNP1313 and 3,4-dichloroaniline (3,4-DCA) (data are presented as mean values with standard deviation). The fractions are designated as Fxy, where x represents the MeOH concentration in eluting solvent and y indicates the fraction number eluted with the solvent (for example, F20%2 stands for second fraction eluted with 20% MeOH); Figure S6: Relative cell viability of SiHa cells exposed to flash chromatography fractions from *Pseudanabaena galeata* CCNP1313 and 3,4-dichloroaniline (3,4-DCA) (data are presented as mean values with standard deviation). The fractions are designated as Fxy, where x represents the MeOH concentration in eluting solvent and y indicates the fraction number eluted with the solvent (for example, F20%2 stands for second fraction eluted with 20% MeOH); Figure S7: Relative cell viability of T47D cells exposed to flash chromatography fractions from *Pseudanabaena galeata* CCNP1313 and 3,4-dichloroaniline (3,4-DCA) (data are presented as mean values with standard deviation). The fractions are designated as Fxy, where x represents the MeOH concentration in eluting solvent and y indicates the fraction number eluted with the solvent (for example, F20%2 stands for second fraction eluted with 20% MeOH); Figure S8: Dose–response curves for flash chromatography fractions from *Pseudanabaena galeata* CCNP1313 measured by MTT assay in C33A, CaSki, HDFa, HeLa, PC3, SiHa, and T47D cell lines. The fractions are designated as Fx.y, where x represents the MeOH concentration in eluting solvent and y indicates the fraction number eluted with the solvent (for example, F20%.2 stands for second fraction eluted with 20% MeOH); Figure S9: Heatmap of the top 30 LC–MS features correlated with cytotoxicity of *Pseudanabaena galeata* CCNP1313 chromatographic fractions against C-33A cells. Columns show features (*m*/*z*), rows represent fractions, and colour indicates log_10_ intensity (white–red). Left annotations display cell viability (%) of each of the fractions. The fractions are designated as xy, where x represents the MeOH concentration in eluting solvent and y indicates the fraction number eluted with the solvent (for example, 20%2 stands for second fraction eluted with 20% MeOH); Figure S10: Heatmap of the top 30 LC–MS features correlated with cytotoxicity of *Pseudanabaena galeata* CCNP1313 chromatographic fractions against CaSki cells. Columns show features (*m*/*z*), rows represent fractions, and colour indicates log_10_ intensity (white–red). Left annotations display cell viability (%) of each of the fractions. The fractions are designated as xy, where x represents the MeOH concentration in eluting solvent, and y indicates the fraction number eluted with the solvent (for example, 20%2 stands for second fraction eluted with 20% MeOH); Figure S11: Heatmap of the top 30 LC–MS features correlated with cytotoxicity of *Pseudanabaena galeata* CCNP1313 chromatographic fractions against HDFa cells. Columns show features (*m*/*z*), rows represent fractions, and colour indicates log_10_ intensity (white–red). Left annotations display cell viability (%) of each of the fractions. The fractions are designated as xy, where x represents the MeOH concentration in eluting solvent and y indicates the fraction number eluted with the solvent (for example, 20%2 stands for second fraction eluted with 20% MeOH); Figure S12: Heatmap of the top 30 LC–MS features correlated with cytotoxicity of *Pseudanabaena galeata* CCNP1313 chromatographic fractions against HeLa cells. Columns show features (*m*/*z*), rows represent fractions, and colour indicates log_10_ intensity (white–red). Left annotations display cell viability (%) of each of the fractions. The fractions are designated as xy, where x represents the MeOH concentration in eluting solvent and y indicates the fraction number eluted with the solvent (for example, 20%.2 stands for second fraction eluted with 20% MeOH); Figure S13: Heatmap of the top 30 LC–MS features correlated with cytotoxicity of *Pseudanabaena galeata* CCNP1313 chromatographic fractions against PC3 cells. Columns show features (*m*/*z*), rows represent fractions, and colour indicates log_10_ intensity (white–red). Left annotations display cell viability (%) of each of the fractions. The fractions are designated as xy, where x represents the MeOH concentration in eluting solvent and y indicates the fraction number eluted with the solvent (for example, 20%2 stands for second fraction eluted with 20% MeOH); Figure S14: Heatmap of the top 30 LC–MS features correlated with cytotoxicity of *Pseudanabaena galeata* CCNP1313 chromatographic fractions against SiHa cells. Columns show features (*m*/*z*), rows represent fractions, and colour indicates log_10_ intensity (white–red). Left annotations display cell viability (%) of each of the fractions. The fractions are designated as xy, where x represents the MeOH concentration in eluting solvent and y indicates the fraction number eluted with the solvent (for example, 20%2 stands for second fraction eluted with 20% MeOH); Figure S15: Heatmap of the top 30 LC–MS features correlated with cytotoxicity of *Pseudanabaena galeata* CCNP1313 chromatographic fractions against T47D cells. Columns show features (*m*/*z*), rows represent fractions, and colour indicates log_10_ intensity (white–red). Left annotations display cell viability (%) of each of the fractions. The fractions are designated as xy, where x represents the MeOH concentration in eluting solvent and y indicates the fraction number eluted with the solvent (for example, 20%2 stands for second fraction eluted with 20% MeOH); Figure S16: Molecular networking generated on the GNPS platform, where node size corresponds to the precursor intensity, node colour represents the Spearman correlation (blue—negative, white—zero, red—strong positive), and the node border indicates significance after Bonferroni’s multiple testing correction (yellow—positive, dark—negative).

## Abbreviations

The following abbreviations are used in this manuscript:

2-MIB	2-methylisoborneol
A549	Lung cancer cell line
ADCs	Antibody–drug conjugates
BALL-1	Acute lymphoblastoid leukaemia cell line
BGCs	Biosynthetic gene clusters
BxPC-3	Pancreatic cancer cell line
C33-A	Cervical cancer cell line
CaSki	Cervical cancer cell line
DAG	Diacylglycerol
DGDG	Digalactosyldiacylglycerol
DoTC2	Cervical cancer cell line
ESI	Electrospray ionisation
FA	Fatty acid
fastDDA	Fast data-dependent acquisition
FBMN	Feature-Based Molecular Networking
GNPS	Global Natural Products Social Molecular Networking
GP	Galeapeptin
HCoV-OC43	Human coronavirus OC43
HCT116	Colon cancer cell line
HDFa	Adult human dermal fibroblasts
HeLa	Cervical cancer cell line
HL60	Promyelocytic leukaemia cell line
HRMS	High-resolution mass spectrometer
HSV-2	Herpes simplex virus type 2
*m*/*z*	Mass to charge ratio
MAG	Monoacylglycerol
MCF-7	Breast cancer cell line
MeOH	Methanol
MGDG	Monogalactosyldiacylglycerol
MiaPaCa-2	Pancreatic cancer cell line
MTT	3-(4,5-dimethylthiazol-2-yl)-2,5-diphenyltetrazolium-bromide
NUGC-3	Stomach cancer cell line
PANC-1	Pancreatic cancer cell line
PC3	Prostate cancer cell line
PGs	*Pseudanabaena galeata* peptides
SARS-CoV-2	Severe acute respiratory syndrome coronavirus 2
SiHa	Cervical cancer cell line
SQDG	Sulfoquinovosyl diacylglycerol
T47D	Breast cancer cell line
WNV	West Nile Virus
