# Supplementary material for: Cytotoxic Activity of the Baltic Cyanobacterium Pseudanabaena galeata CCNP1313
Source: Toxins (Basel). 2025 Dec 6;17(12):586. doi: 10.3390/toxins17120586 (PMC12737398; doi:10.3390/toxins17120586)
Supplement: Supplementary file 1 [file toxins-17-00586-s001.zip › toxins-3990923-supplementary.pdf]

Article

Supplementary Materials: Cytotoxic Activity of the Baltic Cyanobacterium Pseudanabaena galeata CCNP1313

Marta Cegłowska, Robert Konkel and Hanna Mazur-Marzec

Materials and Methods

Text S1. Bioactivity-Based Molecular Networking Analyses

```
## 1) Input
activity_column <- "ACTIVITY_C-33A"
quant       <- read.csv("20250917_quant.csv", stringsAsFactors = FALSE, check.names = FALSE)
activity <- read.csv("activity.csv", sep = ";", stringsAsFactors = FALSE, check.names = FALSE)
## 2) Viability → Inhibition
cols_act <- grep("^ACTIVITY_", names(activity), value = TRUE)
for (col in cols_act) {
   activity[[col]] <- 100 - as.numeric(activity[[col]]) }
## 3) Quantitative data
sample_cols <- grep("mzML Peak area", colnames(quant), value = TRUE)
tab <- t(quant[, sample_cols])
tab <- data.frame(Sample_name = sub("\\.mzML Peak area", "", rownames(tab)), tab)
rownames(tab) <- NULL
colnames(tab)[-1] <- quant$`row ID`
## 4) Merge with activity
activity_sub <- activity[, c("filename", activity_column)]
colnames(activity_sub) <- c("Sample_name", "Activity")
data_raw <- merge(activity_sub, tab, by = "Sample_name")
data_raw <- data_raw[!is.na(data_raw$Activity), ]
## 5) Log10 intensities
data_log <- data_raw
data_log[,-c(1:2)] <- log10(data_log[,-c(1:2)] + 1)
## 6) Correlations
feature_ids <- colnames(data_log)[-c(1:2)]
ct <- t(sapply(3:ncol(data_log), function(x) {
   y <- data_log[, x]; act <- data_log$Activity
   pearson    <- suppressWarnings(cor.test(scale(act)[,1], scale(y)[,1], method = "pearson"))
   spearman <- suppressWarnings(cor.test(act, y, method = "spearman"))
   c(cor_pearson=pearson$estimate, p_pearson=pearson$p.value,
      cor_spearman=spearman$estimate, p_spearman=spearman$p.value) }))
ct <- as.data.frame(ct); rownames(ct) <- feature_ids
## 7) Multiple testing correction (only positive correlations)
ct$agreement <- mapply(function(cp, cs) ifelse(cp>0 & cs>0,"both_positive","other"),
                                   as.numeric(ct$cor_pearson), as.numeric(ct$cor_spearman))
mask <- ct$agreement=="both_positive"
ct$bonferroni_pos <- ifelse(mask, p.adjust(ct$p_spearman, "bonferroni"), 1)
ct$fdr_BH_pos         <- ifelse(mask, p.adjust(ct$p_spearman, "BH"), 1)
```

```r
results <- data.frame(row_ID=rownames(ct), ct)
## 8) Heatmaps top 30
if (!requireNamespace("ComplexHeatmap", quietly = TRUE)) {
  install.packages("BiocManager"); BiocManager::install("ComplexHeatmap") }
if (!requireNamespace("circlize", quietly = TRUE)) {
  install.packages("circlize") }
library(ComplexHeatmap); library(circlize); library(grid)
make_heatmap <- function(results_sorted, label) {
  top30 <- head(results_sorted$row_ID, 30)
  have_cols <- intersect(top30, colnames(data_raw))
  if (length(have_cols)==0) return()
  mat <- as.matrix(log10(data_raw[, have_cols, drop=FALSE] + 1))
  rownames(mat) <- data_raw$Sample_name
  colnames(mat) <- round(quant$`row m/z`[match(have_cols, quant$`row ID`)], 4)
  rng <- range(mat, na.rm=TRUE); mid <- mean(rng)
  col_fun <- circlize::colorRamp2(c(0, mid, rng[2]), c("white","yellow","red"))
  line_name <- sub("^ACTIVITY_", "", activity_column)
  ht <- Heatmap(mat, name="log10 intensity", col=col_fun,
                cluster_rows=FALSE, cluster_columns=TRUE,
                show_row_names=TRUE, show_column_names=TRUE,
                column_title=paste("Top 30 features correlated with activity against", line_name))
  tiff_filename <- paste0("heatmap_top30_", activity_column, "_", label, ".tiff")
  tiff(tiff_filename, width=4200, height=2800, res=300, compression="lzw")
  draw(ht, heatmap_legend_side="bottom")
  dev.off() }
results_sorted_BH    <- results[order(results$fdr_BH_pos, results$p_spearman), ]
results_sorted_Bonf <- results[order(results$bonferroni_pos, results$p_spearman), ]
make_heatmap(results_sorted_BH, "BH")
make_heatmap(results_sorted_Bonf, "Bonferroni")
```

Results

Table S1. Inhibitory effects on cancer (C-33A, CaSki, HeLa, PC3, SiHa, T47D) and healthy (HDFa) cells exposed to flash chromatography fractions from *Pseudanabaena galeata* CCNP1313. The fractions are designated as Fx.y, where x represents the MeOH concentration in eluting solvent, and y indicates the fraction number eluted with the solvent (for example, F20%.2 stands for second fraction eluted with 20% MeOH). Data are presented as mean IC_{50} values (μg mL^{-1}) with standard deviation, 95% confidence intervals, and selectivity index for fraction F20%.1.

**Sample**	**IC_{50} [μg mL^{-1}]**						
	**C-33A**	**CaSki**	**HDFa**	**HeLa**	**PC3**	**SiHa**	**T47D**
**F20%.1**	-	-	66 ± 5<br>(53-79)	83 ± 7<br>(68-99)<br>SI = 0.795	-	119 ± 23<br>(61-176)<br>SI = 0.554	-
**F20%.2**	-	-	-	-	-	-	-
**F30%.1**	-	-	-	-	-	-	-
**F30%.2**	-	-	-	-	-	-	-
**F40%.1**	-	-	-	-	-	-	-
**F40%.2**	-	-	-	-	-	-	-
**F50%.1**	-	-	-	-	-	-	-
**F50%.2**	-	-	-	-	-	-	-
**F50%.3**	-	-	-	-	-	-	-
**F60%.1**	-	-	-	-	-	-	-
**F60%.2**	-	-	-	-	-	-	-
**F60%.3**	-	-	-	-	-	-	-
**F70%.1**	-	-	-	-	-	-	-
**F70%.2**	-	-	-	-	-	-	-
**F70%.3**	-	-	-	-	-	-	200 ± 30<br>(125-274)
**F80%.1**	-	-	-	-	-	-	180 ± 20<br>(129-229)
**F80%.2**	150 ± 23<br>(93-207)	194 ± 29<br>(121-267)	-	-	-	-	173 ± 14<br>(138-208)
**F80%.3**	138 ± 18<br>(94-183)	167 ± 18<br>(124-211)	-	-	-	-	173 ± 15<br>(135-211)
**F80%.4**	142 ± 13<br>(109-175)	150 ± 30<br>(76-225)	-	-	-	-	173 ± 21<br>(122-224)
**F90%.1**	153 ± 13<br>(122-186)	145 ± 23<br>(88-202)	-	-	-	-	172 ± 11<br>(145-200)
**F90%.2**	89 ± 6<br>(74-104)	115 ± 15<br>(76-153)	-	-	-	-	164 ± 14<br>(130-198)
**F90%.3**	129 ± 11<br>(102-157)	112 ± 20<br>(61-162)	-	-	-	140 ± 10<br>(115-165)	107 ± 18<br>(63-150)
**F100%.1**	147 ± 9<br>(126-168)	142 ± 18<br>(97-187)	-	-	-	-	157 ± 23<br>(100-214)
**F100%.2**	121 ± 8<br>(101-141)	84 ± 6<br>(69-99)	-	129 ± 5<br>(113-145)	-	153 ± 15<br>(115-191)	129 ± 15<br>(92-167)
**F100%.3**	95 ± 13<br>(62-128)	100 ± 10<br>(76-124)	-	-	-	136 ± 15<br>(98-173)	110 ± 20<br>(62-159)
**F100%.4**	94 ± 5<br>(81-107)	92 ± 11<br>(65-118)	-	140 ± 14<br>(106-174)	180 ± 20<br>(130-230)	115 ± 9<br>(92-138)	112 ± 14<br>(79-146)

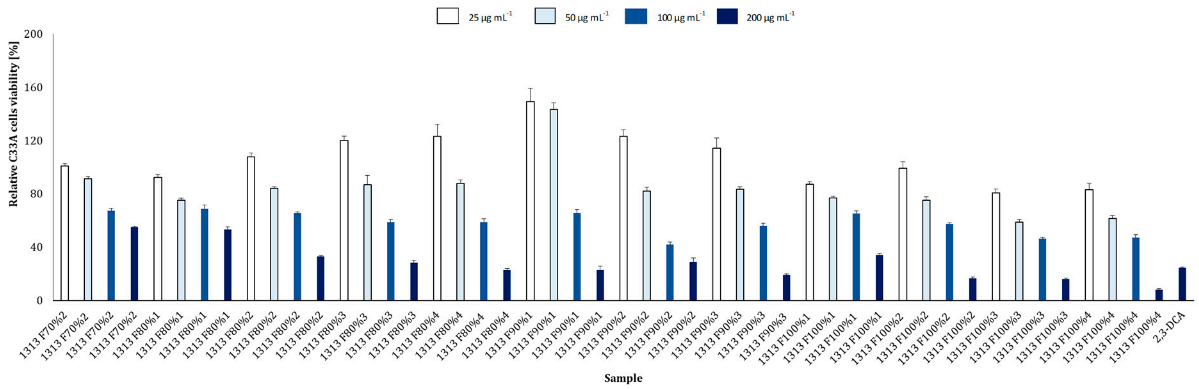

Figure S1. Relative cell viability of C33A cells exposed to flash chromatography fractions from *Pseudanabaena galeata* CCNP1313 and 3,4-dichloroaniline (3,4-DCA) (data are presented as mean values with standard deviation). The fractions are designated as Fxy, where x represents the MeOH concentration in eluting solvent, and y indicates the fraction number eluted with the solvent (for example, F20%2 stands for second fraction eluted with 20% MeOH).

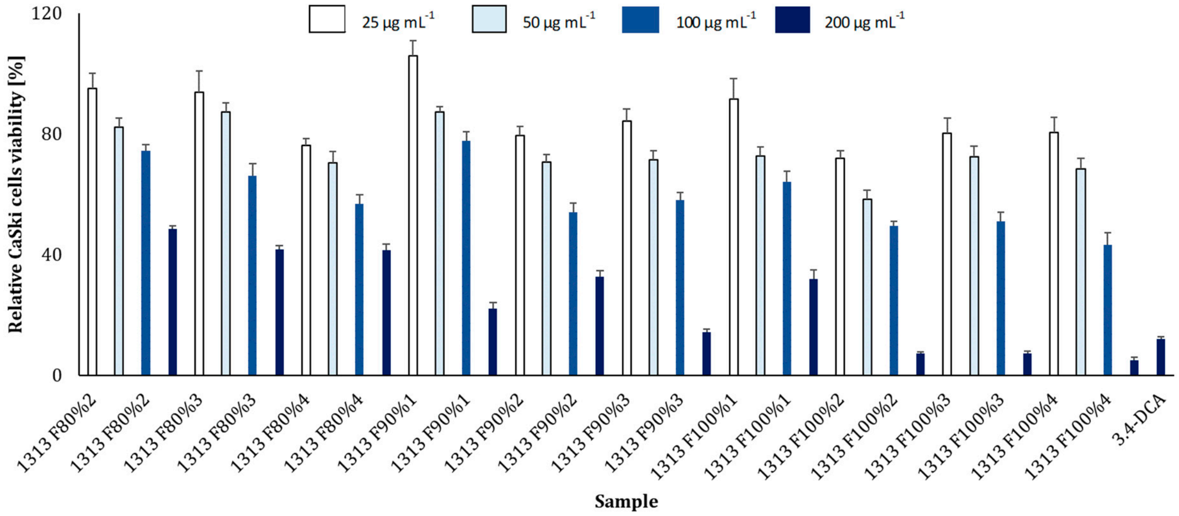

Figure S2. Relative cell viability of CaSki cells exposed to flash chromatography fractions from *Pseudanabaena galeata* CCNP1313 and 3,4-dichloroaniline (3,4-DCA) (data are presented as mean values with standard deviation). The fractions are designated as Fxy, where x represents the MeOH concentration in eluting solvent, and y indicates the fraction number eluted with the solvent (for example, F20%2 stands for second fraction eluted with 20% MeOH).

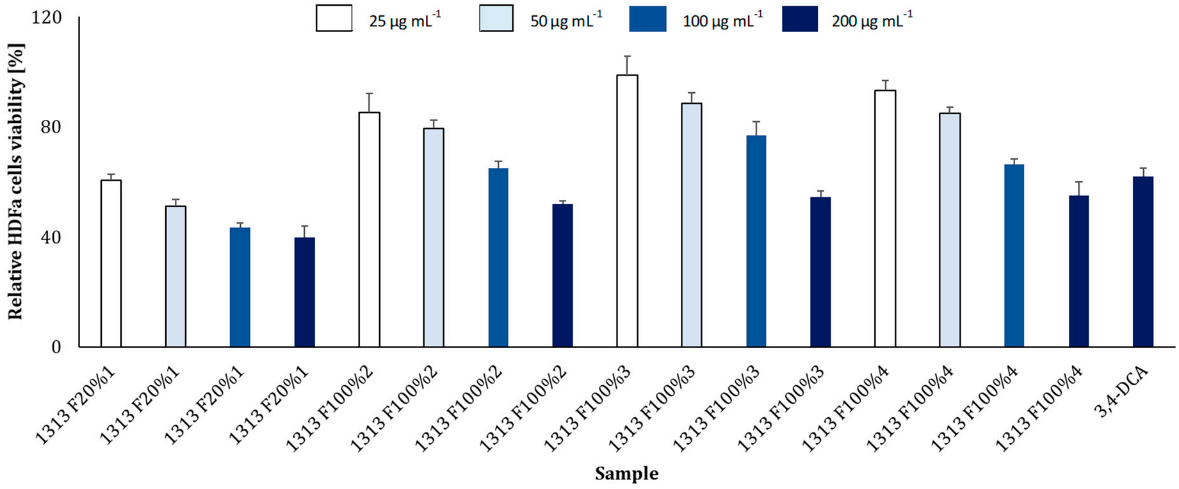

Figure S3. Relative cell viability of HDFa cells exposed to flash chromatography fractions from *Pseudanabaena galeata* CCNP1313 and 3,4-dichloroaniline (3,4-DCA) (data are presented as mean values with standard deviation). The fractions are designated as Fxy, where x represents the MeOH concentration in eluting solvent, and y indicates the fraction number eluted with the solvent (for example, F20%2 stands for second fraction eluted with 20% MeOH).

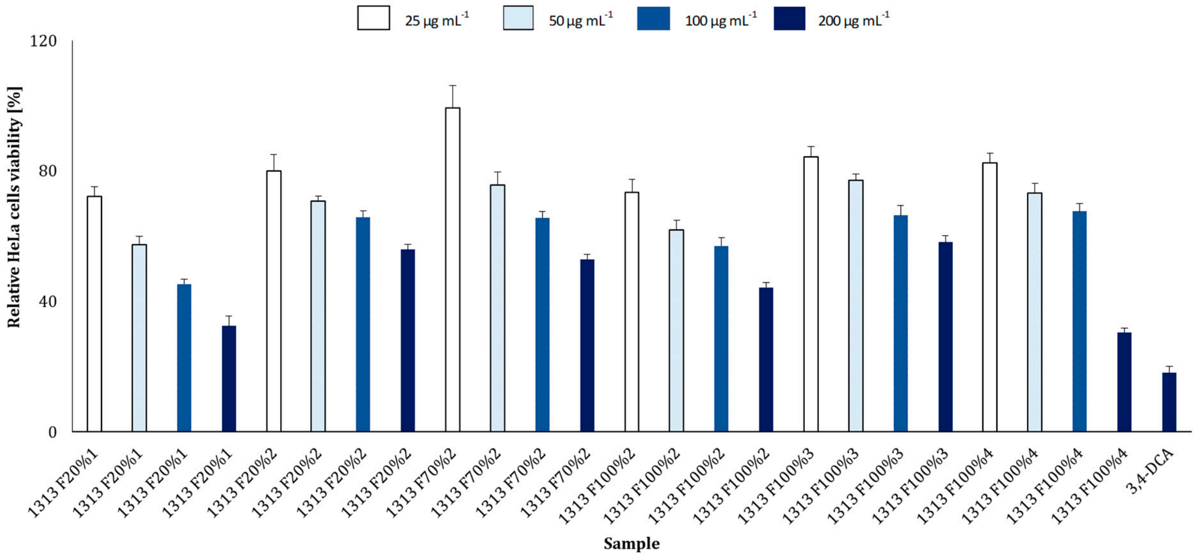

Figure S4. Relative cell viability of HeLa cells exposed to flash chromatography fractions from *Pseudanabaena galeata* CCNP1313 and 3,4-dichloroaniline (3,4-DCA) (data are presented as mean values with standard deviation). The fractions are designated as Fxy, where x represents the MeOH concentration in eluting solvent, and y indicates the fraction number eluted with the solvent (for example, F20%2 stands for second fraction eluted with 20% MeOH).

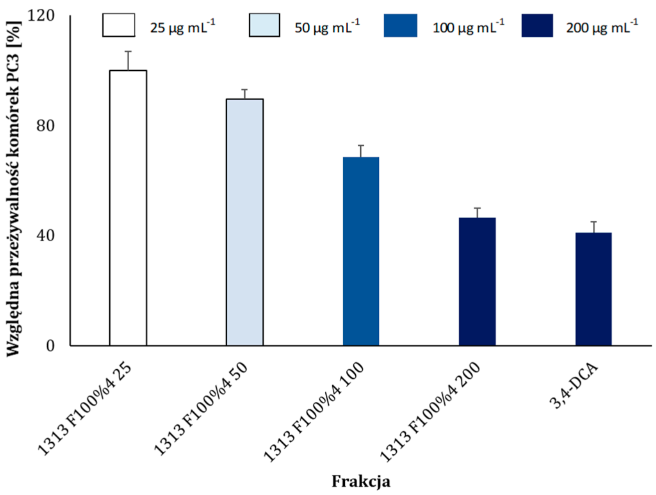

Figure S5. Relative cell viability of PC3 cells exposed to flash chromatography fractions from *Pseudanabaena galeata* CCNP1313 and 3,4-dichloroaniline (3,4-DCA) (data are presented as mean values with standard deviation). The fractions are designated as Fxy, where x represents the MeOH concentration in eluting solvent, and y indicates the fraction number eluted with the solvent (for example, F20%2 stands for second fraction eluted with 20% MeOH).

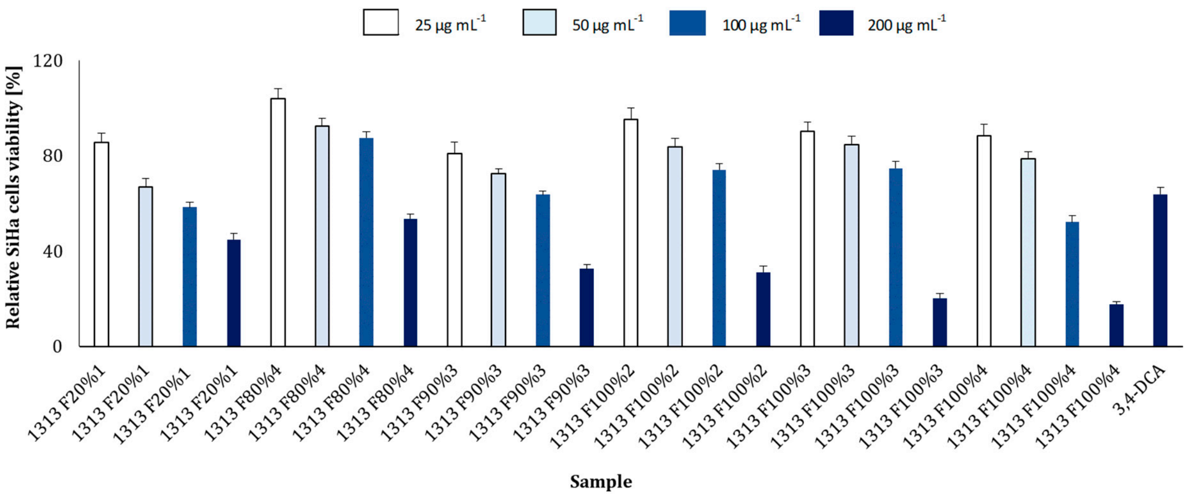

Figure S6. Relative cell viability of SiHa cells exposed to flash chromatography fractions from *Pseudanabaena galeata* CCNP1313 and 3,4-dichloroaniline (3,4--DCA) (data are presented as mean values with standard deviation). The fractions are designated as Fxy, where x represents the MeOH concentration in eluting solvent, and y indicates the fraction number eluted with the solvent (for example, F20%2 stands for second fraction eluted with 20% MeOH).

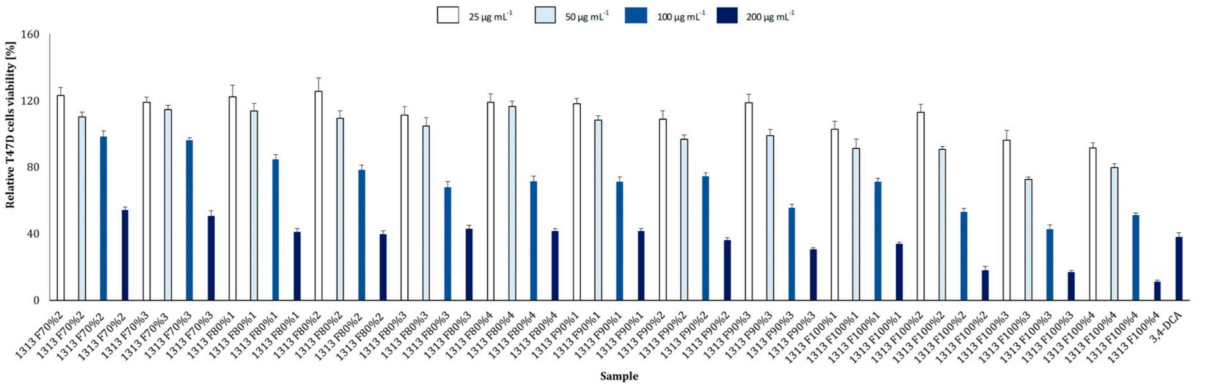

Figure S7. Relative cell viability of T47D cells exposed to flash chromatography fractions from *Pseudanabaena galeata* CCNP1313 and 3,4-dichloroaniline (3,4-DCA) (data are presented as mean values with standard deviation). The fractions are designated as Fxy, where x represents the MeOH concentration in eluting solvent, and y indicates the fraction number eluted with the solvent (for example, F20%2 stands for second fraction eluted with 20% MeOH).

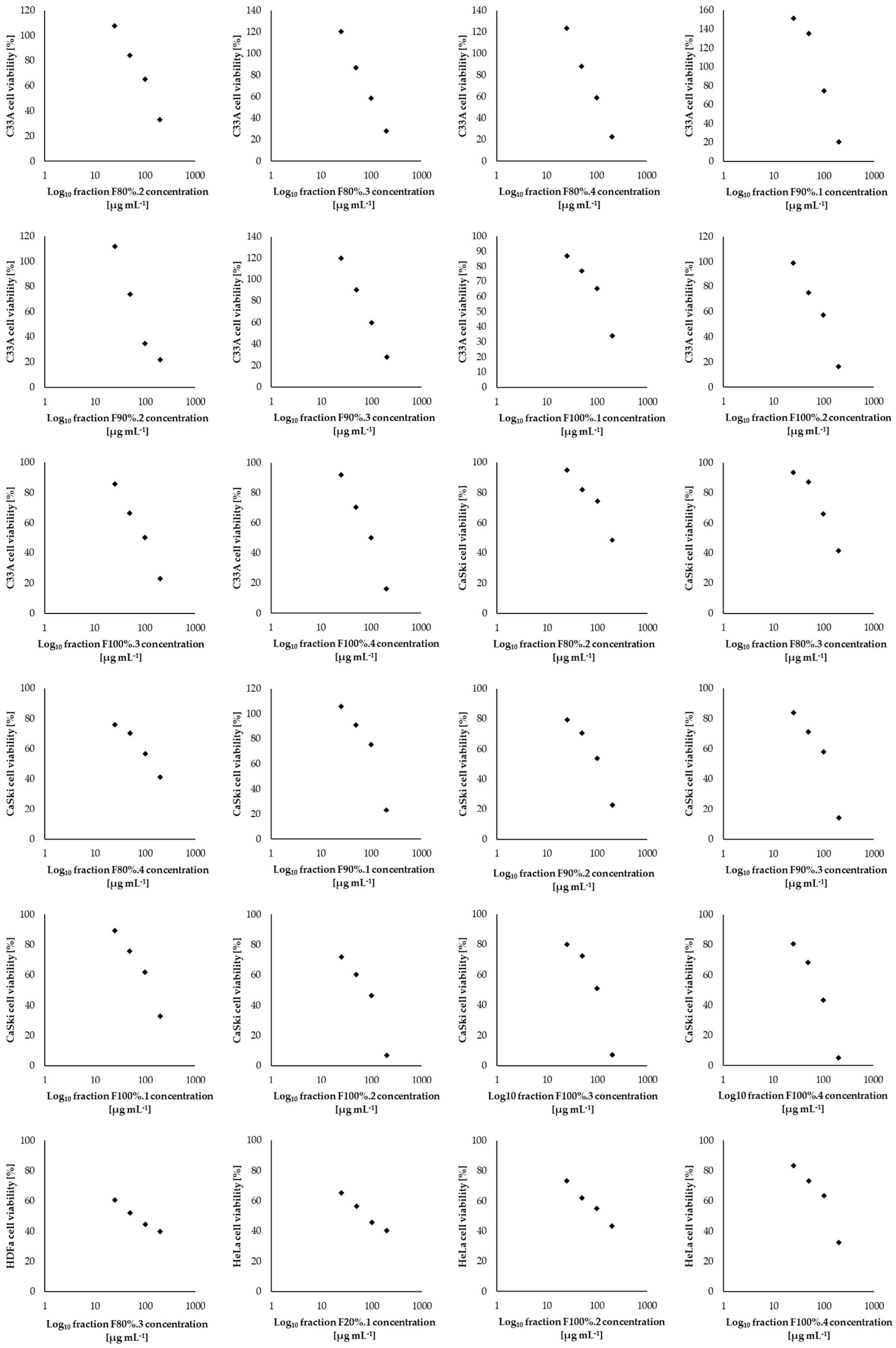

C33A cell viability [%]
Log10 fraction F80%.2 concentration [μg mL-1]
C33A cell viability [%]
Log10 fraction F80%.3 concentration [μg mL-1]
C33A cell viability [%]
Log10 fraction F80%.4 concentration [μg mL-1]
C33A cell viability [%]
Log10 fraction F90%.1 concentration [μg mL-1]
C33A cell viability [%]
Log10 fraction F90%.2 concentration [μg mL-1]
C33A cell viability [%]
Log10 fraction F90%.3 concentration [μg mL-1]
C33A cell viability [%]
Log10 fraction F100%.1 concentration [μg mL-1]
C33A cell viability [%]
Log10 fraction F100%.2 concentration [μg mL-1]
C33A cell viability [%]
Log10 fraction F100%.3 concentration [μg mL-1]
C33A cell viability [%]
Log10 fraction F100%.4 concentration [μg mL-1]
CaSki cell viability [%]
Log10 fraction F80%.2 concentration [μg mL-1]
CaSki cell viability [%]
Log10 fraction F80%.3 concentration [μg mL-1]
CaSki cell viability [%]
Log10 fraction F80%.4 concentration [μg mL-1]
CaSki cell viability [%]
Log10 fraction F90%.1 concentration [μg mL-1]
CaSki cell viability [%]
Log10 fraction F90%.2 concentration [μg mL-1]
CaSki cell viability [%]
Log10 fraction F90%.3 concentration [μg mL-1]
CaSki cell viability [%]
Log10 fraction F100%.1 concentration [μg mL-1]
CaSki cell viability [%]
Log10 fraction F100%.2 concentration [μg mL-1]
CaSki cell viability [%]
Log10 fraction F100%.3 concentration [μg mL-1]
CaSki cell viability [%]
Log10 fraction F100%.4 concentration [μg mL-1]
HDFa cell viability [%]
Log10 fraction F80%.3 concentration [μg mL-1]
HeLa cell viability [%]
Log10 fraction F20%.1 concentration [μg mL-1]
HeLa cell viability [%]
Log10 fraction F100%.2 concentration [μg mL-1]
HeLa cell viability [%]
Log10 fraction F100%.4 concentration [μg mL-1]

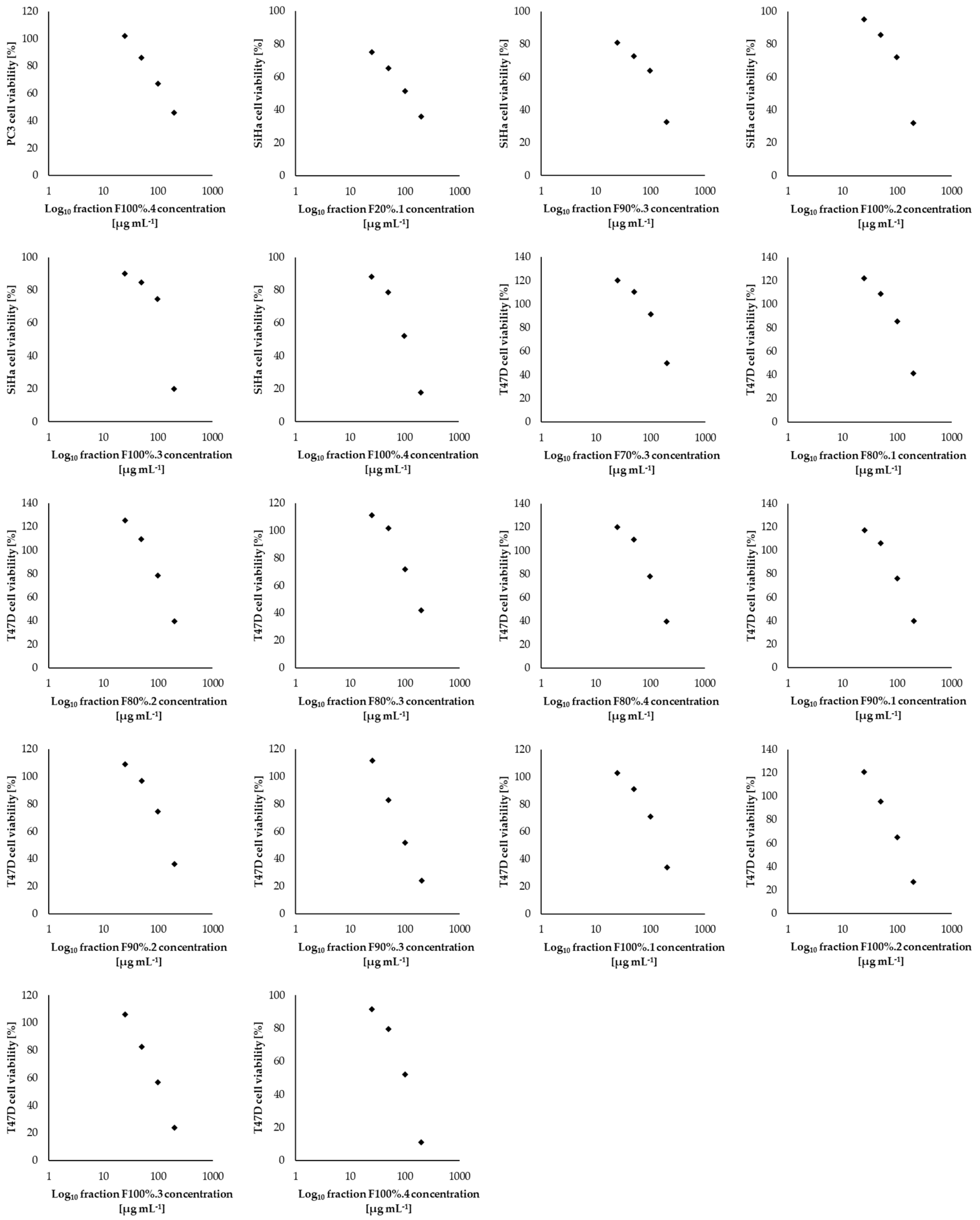

Figure S8. Dose–response curves for flash chromatography fractions from *Pseudanabaena galeata* CCNP1313 measured by MTT assay in C33A, CaSki, HDFa, HeLa, PC3, SiHa, and T47D cell lines. The fractions are designated as Fx.y, where x represents the MeOH concentration in eluting solvent, and y indicates the fraction number eluted with the solvent (for example, F20%.2 stands for second fraction eluted with 20% MeOH).

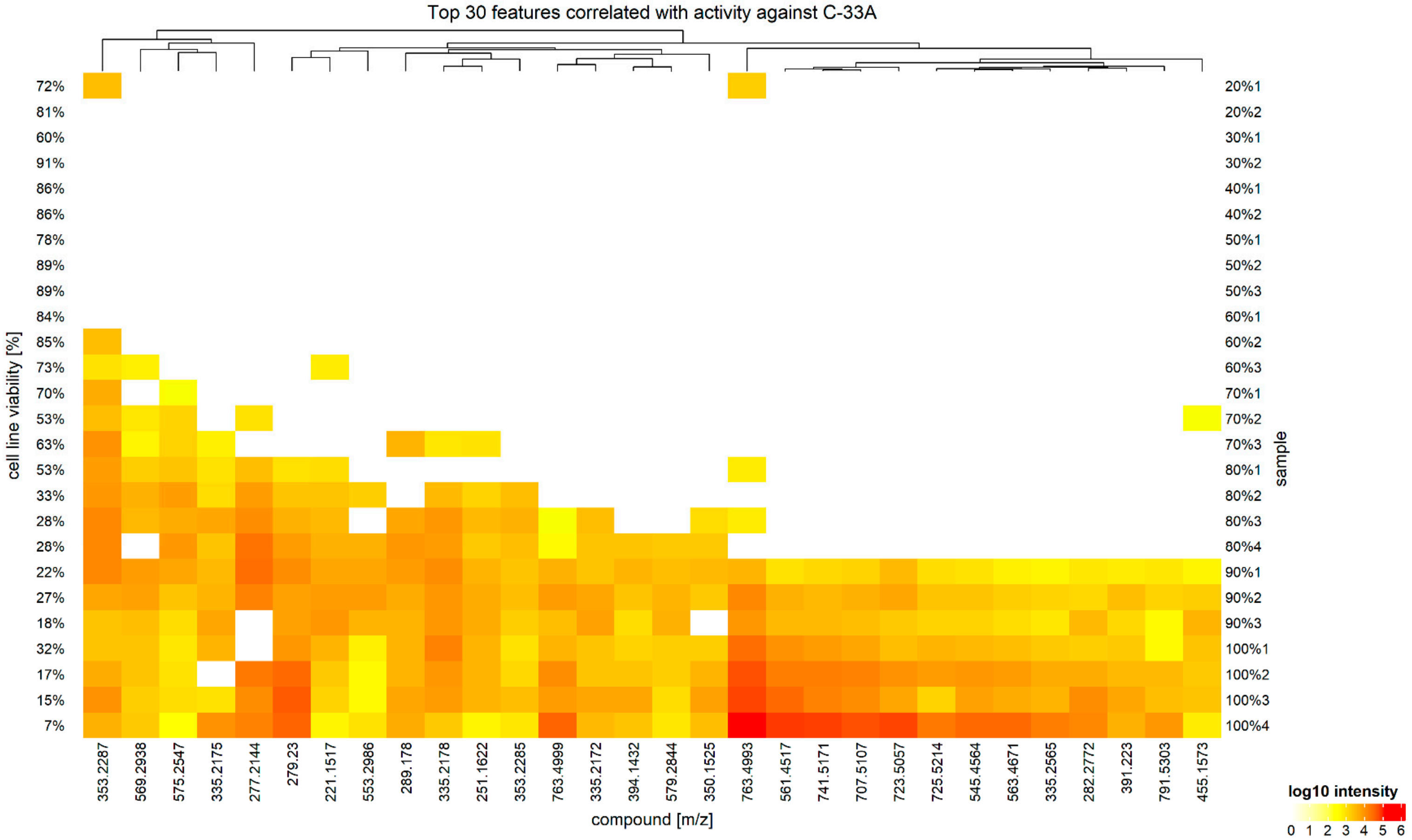

Figure S9. Heatmap of the top 30 LC–MS features correlated with cytotoxicity of *Pseudanabaena galeata* CCNP1313 chromatographic fractions against C-33A cells. Columns show features (*m*/*z*), rows represent fractions, and colour indicates $\log_{10}$ intensity (white–red). Left annotations display cell viability (%) of each of the fractions. The fractions are designated as xy, where x represents the MeOH concentration in eluting solvent, and y indicates the fraction number eluted with the solvent (for example, 20%2 stands for second fraction eluted with 20% MeOH).

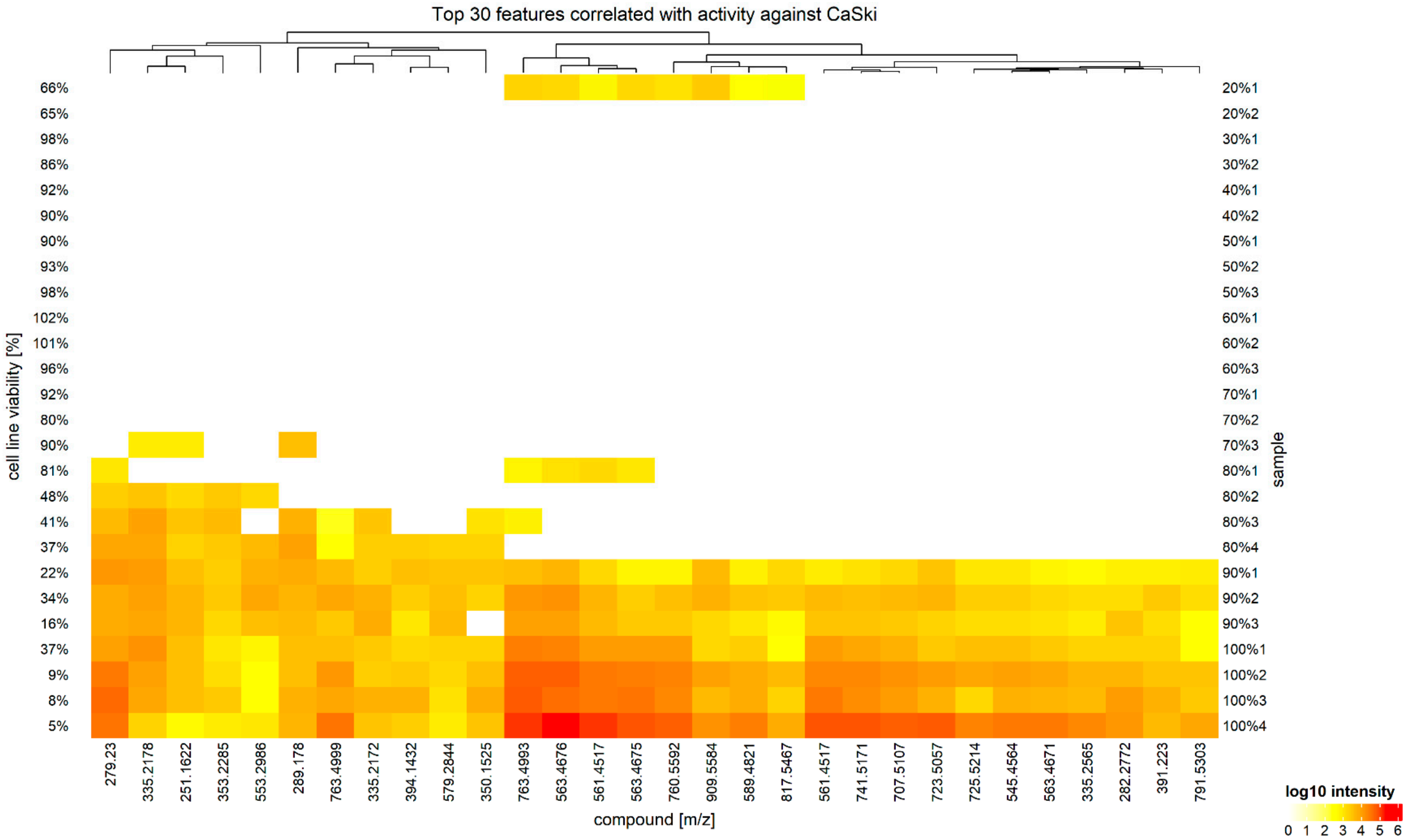

Figure S10. Heatmap of the top 30 LC–MS features correlated with cytotoxicity of *Pseudanabaena galeata* CCNP1313 chromatographic fractions against CaSki cells. Columns show features (*m*/*z*), rows represent fractions, and colour indicates $\log_{10}$ intensity (white–red). Left annotations display cell viability (%) of each of the fractions. The fractions are designated as xy, where x represents the MeOH concentration in eluting solvent, and y indicates the fraction number eluted with the solvent (for example, 20%2 stands for second fraction eluted with 20% MeOH).

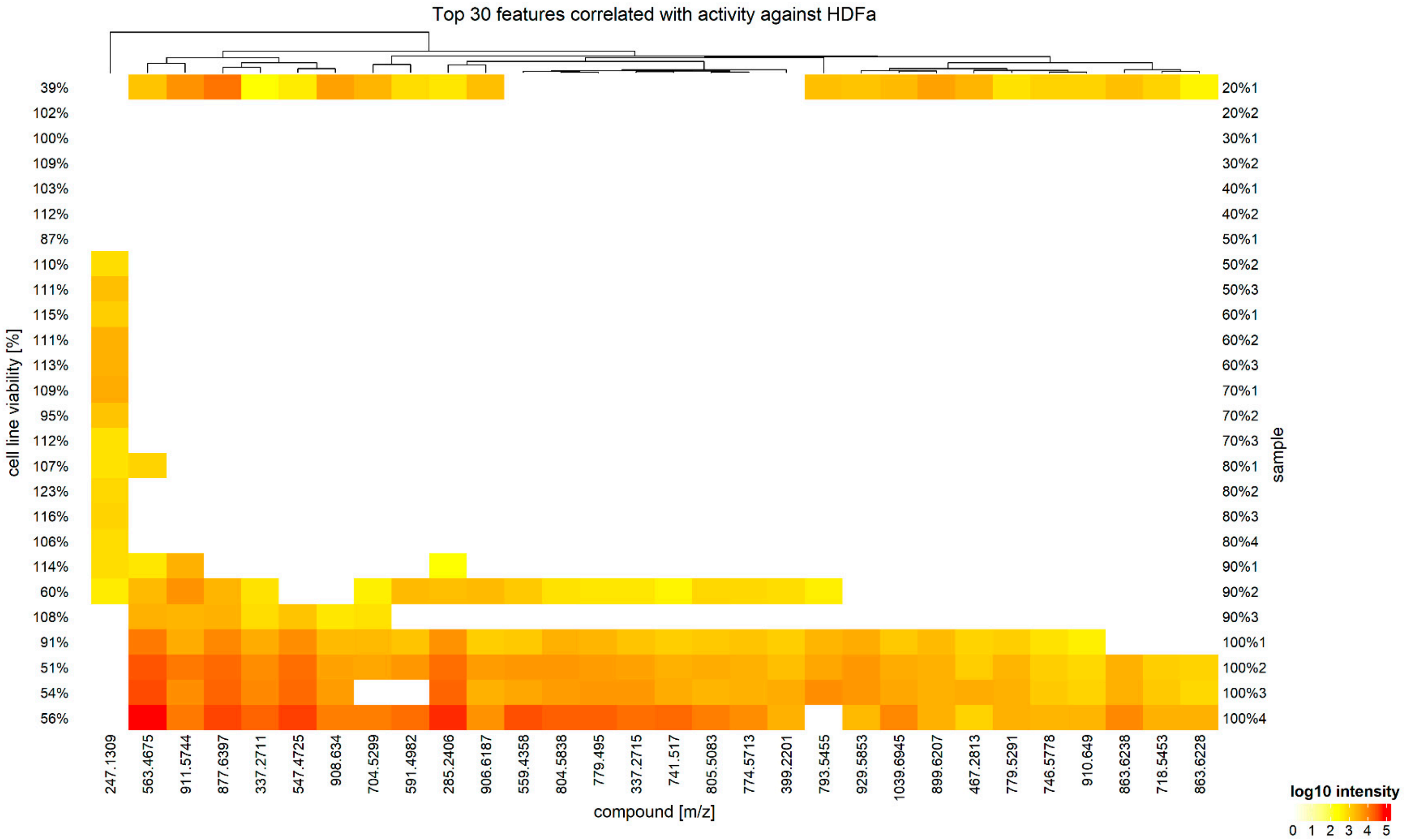

Figure S11. Heatmap of the top 30 LC–MS features correlated with cytotoxicity of *Pseudanabaena galeata* CCNP1313 chromatographic fractions against HDFa cells. Columns show features (*m*/*z*), rows represent fractions, and colour indicates log_{10} intensity (white–red). Left annotations display cell viability (%) of each of the fractions. The fractions are designated as xy, where x represents the MeOH concentration in eluting solvent, and y indicates the fraction number eluted with the solvent (for example, 20%2 stands for second fraction eluted with 20% MeOH).

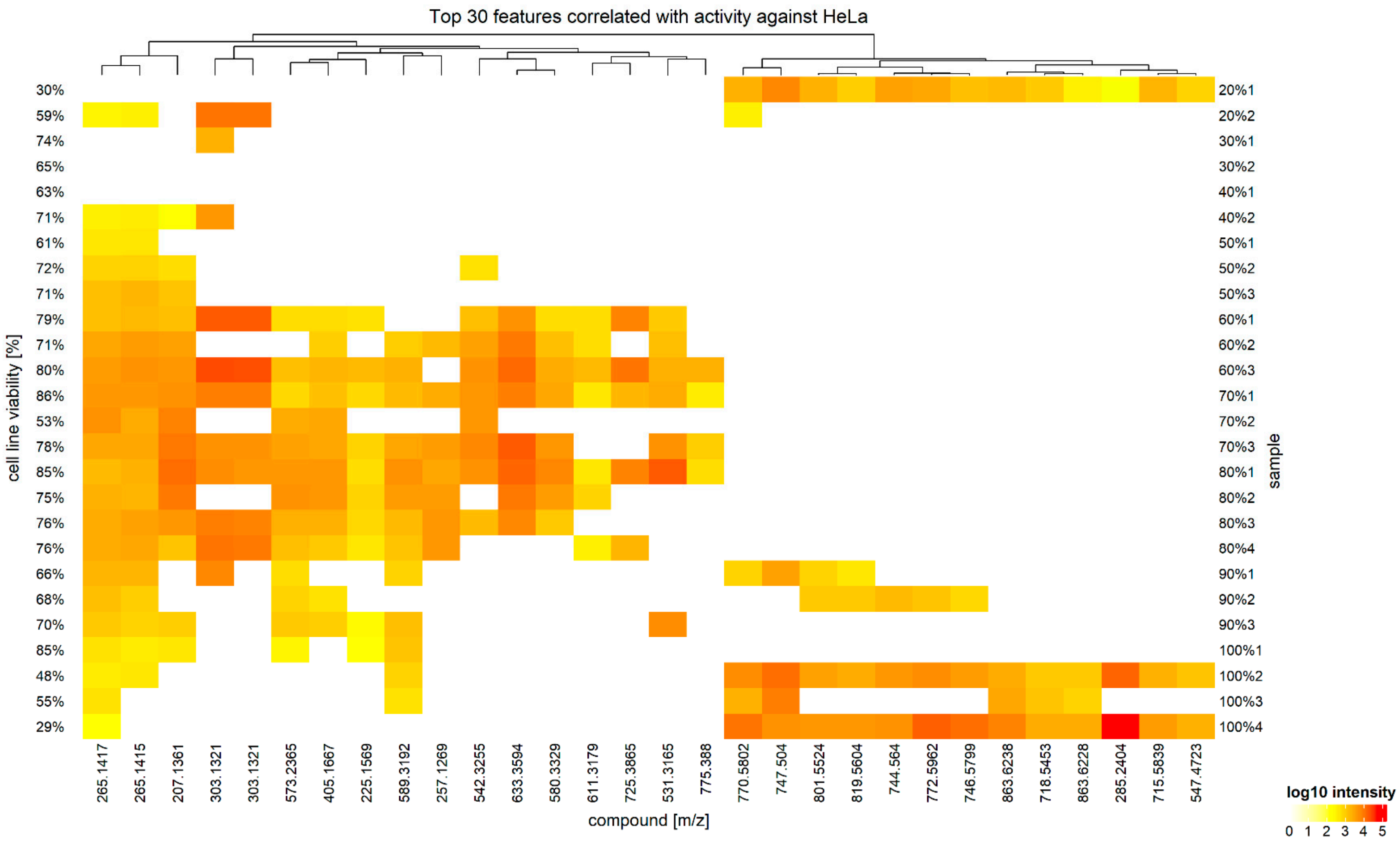

Figure S12. Heatmap of the top 30 LC–MS features correlated with cytotoxicity of *Pseudanabaena galeata* CCNP1313 chromatographic fractions against HeLa cells. Columns show features (*m*/*z*), rows represent fractions, and colour indicates $\log_{10}$ intensity (white–red). Left annotations display cell viability (%) of each of the fractions. The fractions are designated as xy, where x represents the MeOH concentration in eluting solvent, and y indicates the fraction number eluted with the solvent (for example, 20%2 stands for second fraction eluted with 20% MeOH).

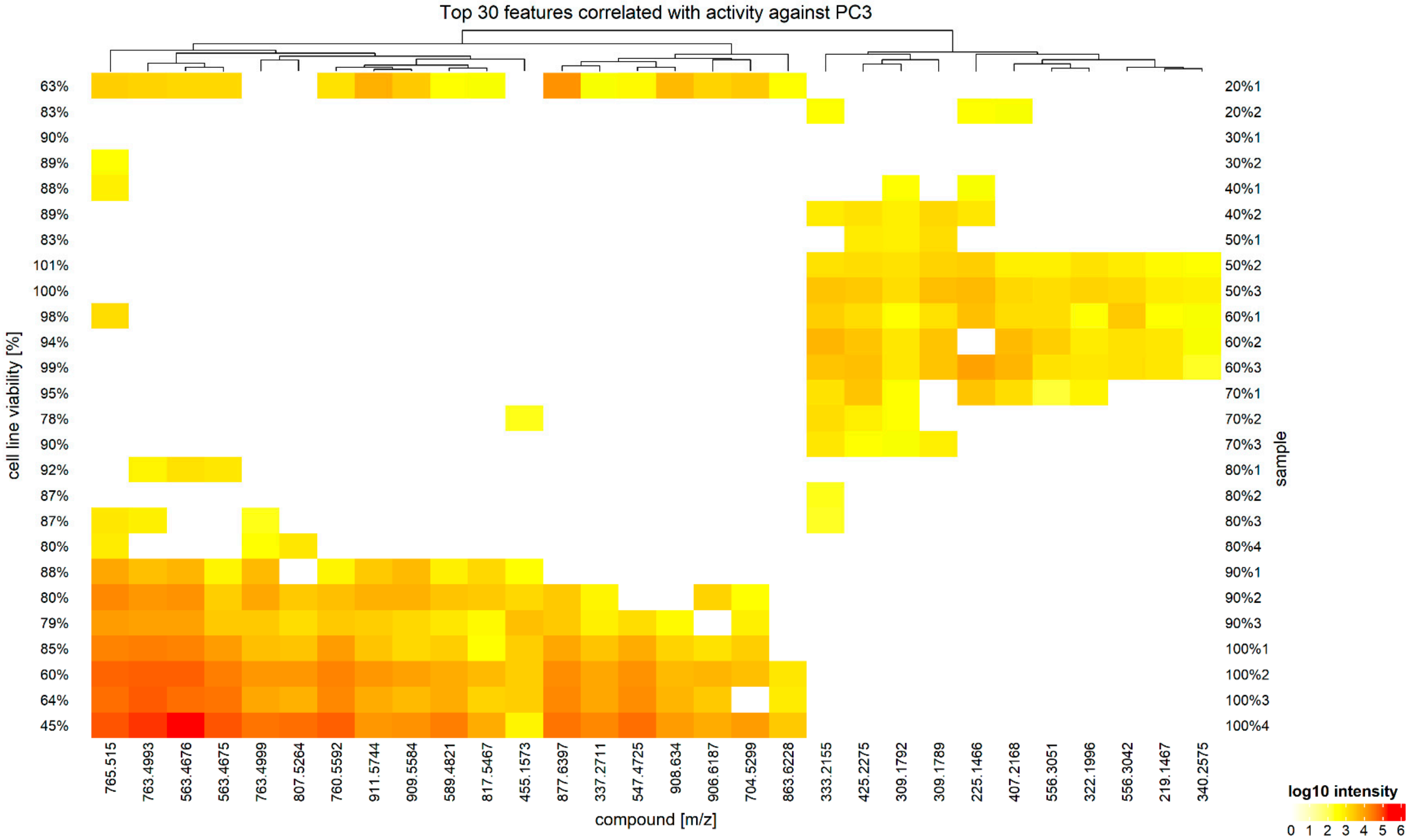

Figure S13. Heatmap of the top 30 LC–MS features correlated with cytotoxicity of *Pseudanabaena galeata* CCNP1313 chromatographic fractions against PC3 cells. Columns show features (*m*/*z*), rows represent fractions, and colour indicates $\log_{10}$ intensity (white–red). Left annotations display cell viability (%) of each of the fractions. The fractions are designated as xy, where x represents the MeOH concentration in eluting solvent, and y indicates the fraction number eluted with the solvent (for example. 20%2 stands for second fraction eluted with 20% MeOH).

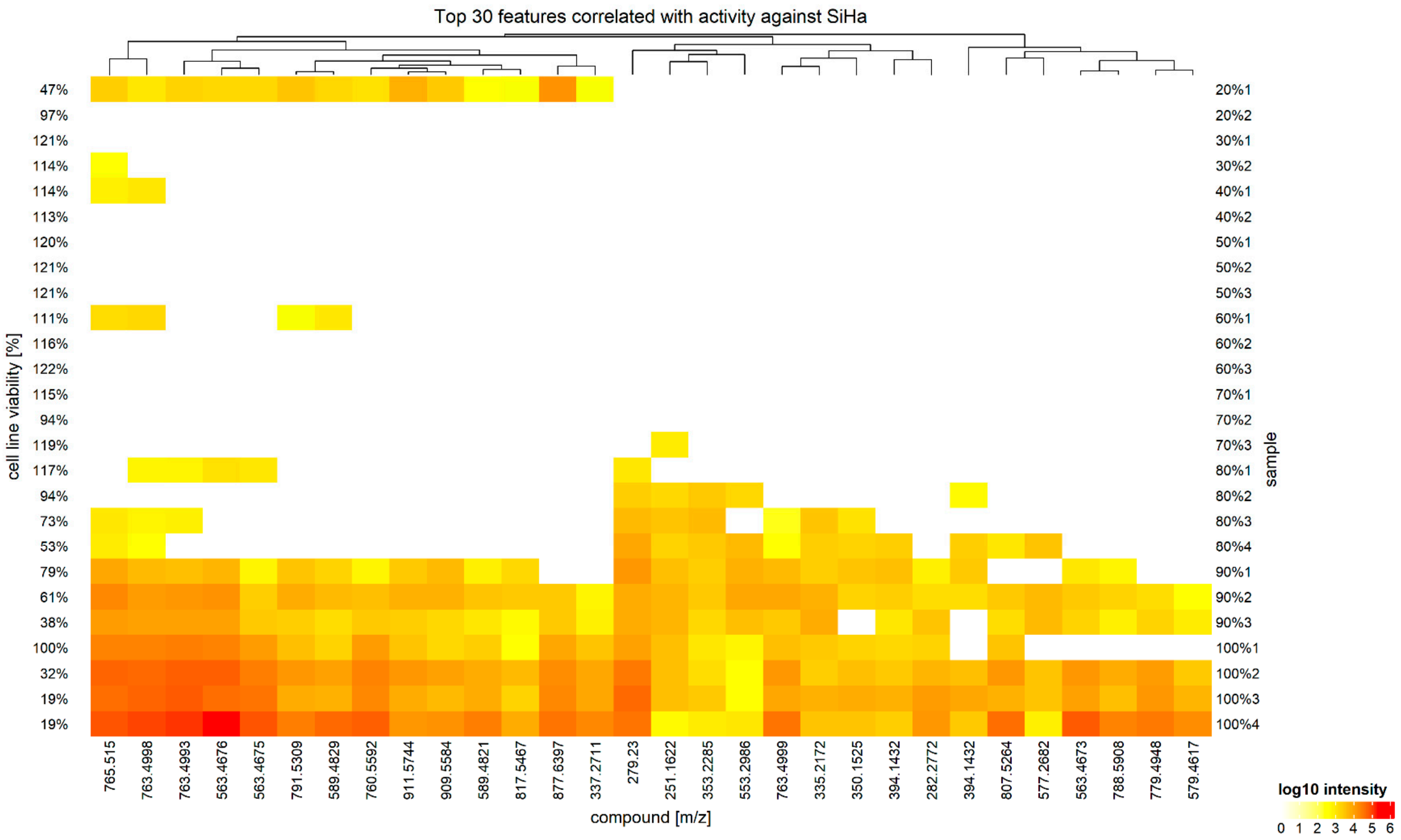

Figure S14. Heatmap of the top 30 LC–MS features correlated with cytotoxicity of *Pseudanabaena galeata* CCNP1313 chromatographic fractions against SiHa cells. Columns show features (*m*/*z*), rows represent fractions, and colour indicates $\log_{10}$ intensity (white–red). Left annotations display cell viability (%) of each of the fractions. The fractions are designated as xy, where x represents the MeOH concentration in eluting solvent, and y indicates the fraction number eluted with the solvent (for example, 20%2 stands for second fraction eluted with 20% MeOH).

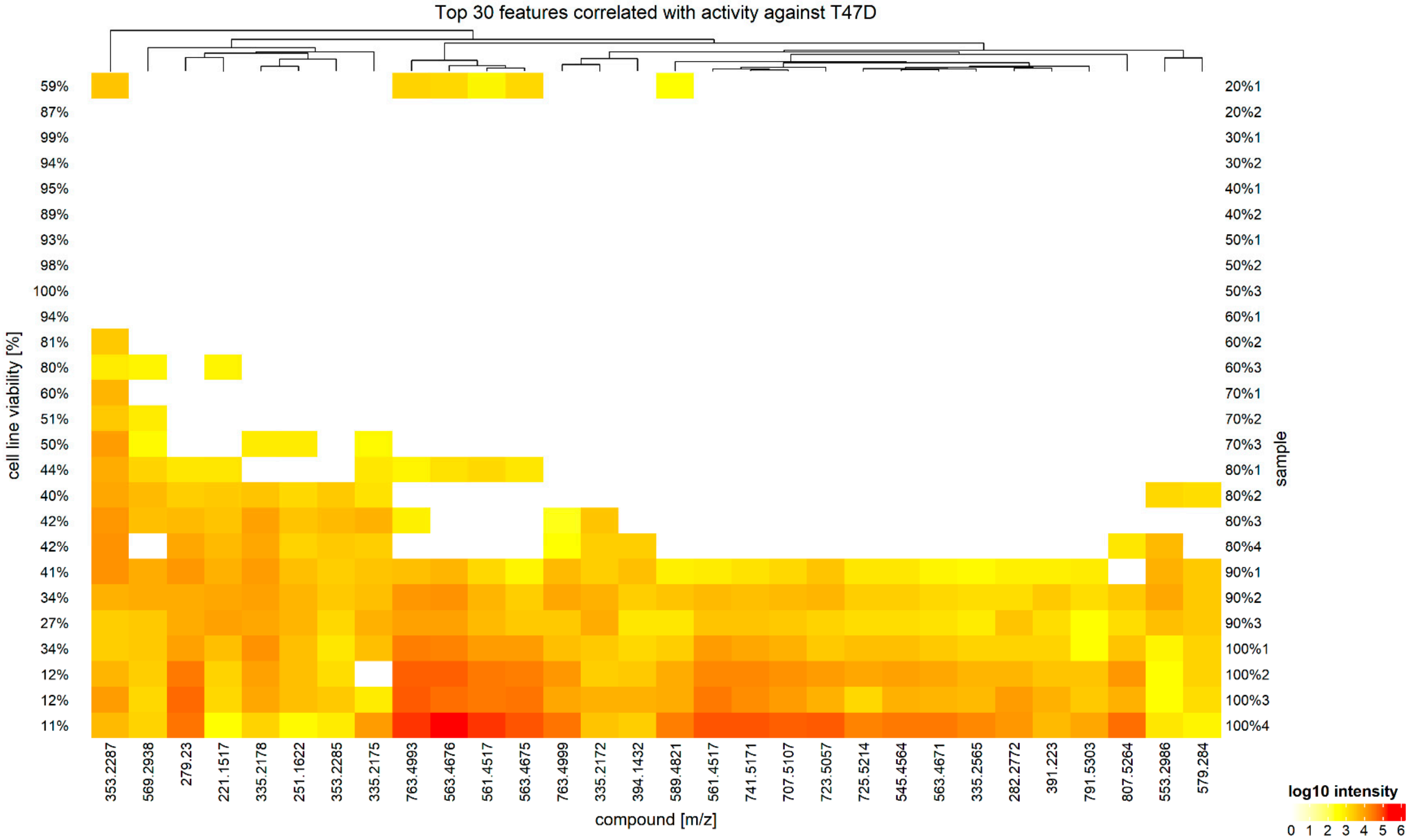

Figure S15. Heatmap of the top 30 LC–MS features correlated with cytotoxicity of *Pseudanabaena galeata* CCNP1313 chromatographic fractions against T47D cells. Columns show features (*m*/*z*), rows represent fractions, and colour indicates $\log_{10}$ intensity (white–red). Left annotations display cell viability (%) of each of the fractions. The fractions are designated as xy, where x represents the MeOH concentration in eluting solvent, and y indicates the fraction number eluted with the solvent (for example 20%2 stands for second fraction eluted with 20% MeOH).

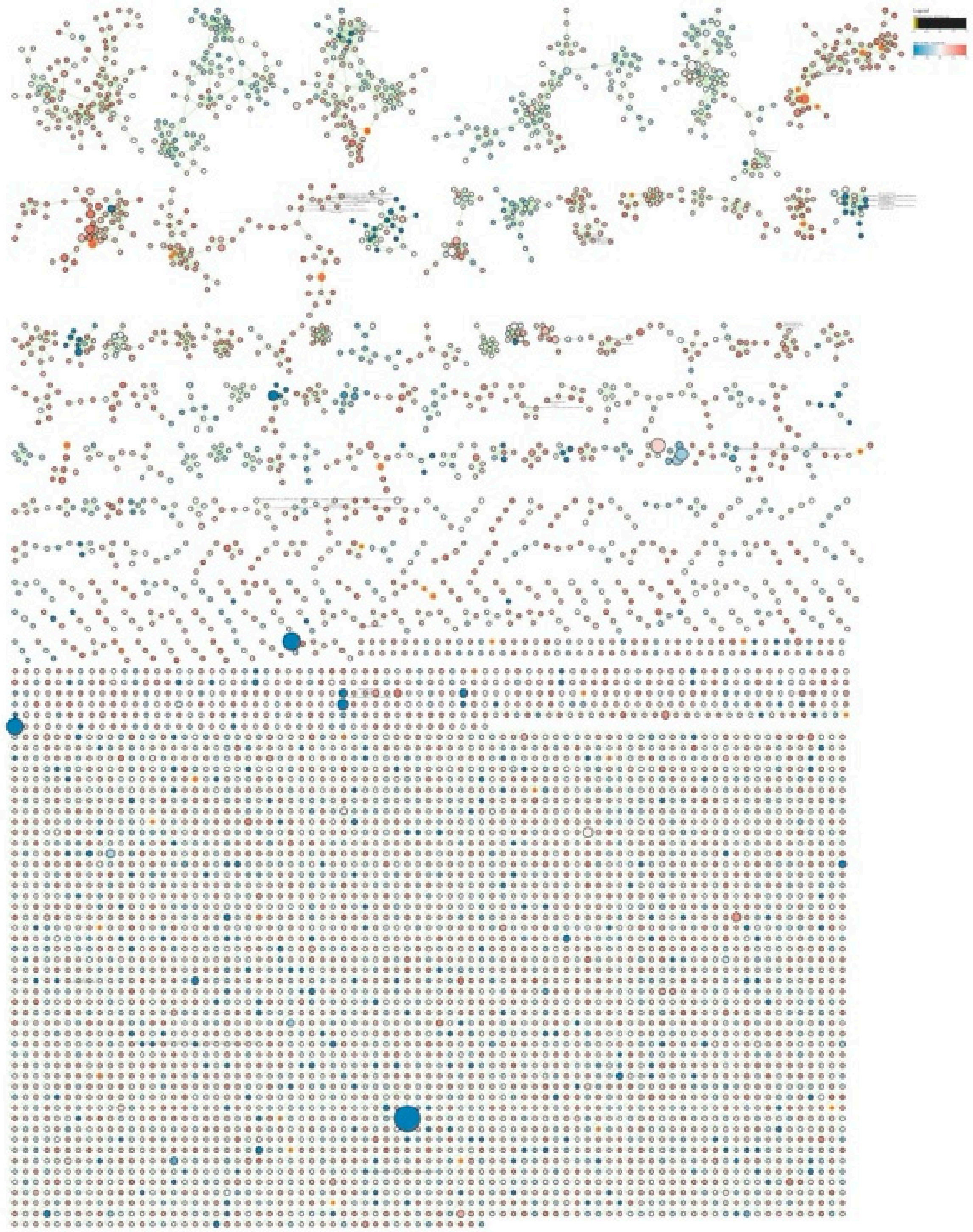

Figure S16. Molecular networking generated on the GNPS platform, where node size corresponds to the precursor intensity, node colour represents the Spearman correlation (blue – negative, white – zero, red – strong positive), and the node border indicates significance after Bonferroni’s multiple testing correction (yellow – positive, dark – negative).
